# General synthesis of ultrafine metal oxide/reduced graphene oxide nanocomposites for ultrahigh-flux nanofiltration membrane

**DOI:** 10.1038/s41467-022-28180-4

**Published:** 2022-01-25

**Authors:** Wanyu Zhang, Hai Xu, Fei Xie, Xiaohua Ma, Bo Niu, Mingqi Chen, Hongyu Zhang, Yayun Zhang, Donghui Long

**Affiliations:** State Key Laboratory of Chemical Engineering, East China University of Science and Technology, Shanghai, 200237 China

**Keywords:** Nanoscale materials, Mechanical and structural properties and devices, Soft materials

## Abstract

Graphene-based membranes have great potential to revolutionize nanofiltration technology, but achieving high solute rejections at high water flux remains extremely challenging. Herein, a family of ultrafine metal oxide/reduced graphene oxide (rGO) nanocomposites are synthesized through a heterogenous nucleation and diffusion-controlled growth process for dye nanofiltration. The synthesis is based on the utilization of oxygen functional groups on GO surface as preferential active sites for heterogeneous nucleation, leading to the formation of sub-3 nm size, monodispersing as well as high-density loading of metal oxide nanoparticles. The anchored ultrafine nanoparticles could inhibit the wrinkling of the rGO nanosheet, forming highly stable colloidal solutions for the solution processing fabrication of nanofiltration membranes. By functioning as pillars, the nanoparticles remarkably increase both vertical interlayer spacing and lateral tortuous paths of the rGO membranes, offering a water permeability of 225 L m^−2^ h^−1^ bar^−1^ and selectivity up to 98% in the size-exclusion separation of methyl blue.

## Abstract (web summary)

Graphene oxide nanofiltration membranes with tunable interlayer spacing tend to be either unstable in water or have low water permeation rates. Here the authors report a general synthetic method to achieve ultrafine metal oxide - reduced graphene oxide nanocomposites for dye filtration, achieving high water permeability and selectivity.

## Introduction

Nanofiltration is an energy-efficient membrane separation process that can reject efficiently multivalent ions and organic compounds, showing great promising in water treatment applications, and particularly in pharmaceutical and food industries^1–4^. Generally, the nanofiltration membranes have nanopores in the range of 0.5–5 nm, which could the fulfill nominal molecular weight cutoff of 200–1000 Da^5,6^. And the separation mechanism is majorly governed by the size-based and charge-based exclusion, which depends strongly both on the membrane structure and on the interaction between the membrane and the solute^7,8^.

Recently, a great advance has been made in the graphene-based membranes for nanofiltration^9^. Graphene is an up-and-coming 2D material with good flexibility, large surface area, excellent electrical conductivity, and high surface activity^10^. It thus has been considered as an ideal membrane material, especially for desalination^11^. More typically, the graphene oxide (GO) composed of graphite-like and oxidized region feature water migration and simultaneously highly efficient molecule sieving^12^. GO is dispersible in water and many other polar solvents, making it be easily fabricated into laminated membrane materials via solution processing techniques for nanofiltration separations^13–18^. The 2D interlayer spacing between GO nanosheets can be used as nanochannels for water permeation while rejecting the larger species^19^. And the interlayer spacing of GO nanosheets can be further adjusted to achieve size-selective molecular sieving^20^. However, GO nanosheets are extremely hydrophilic, causing uncontrollable swelling and poor stability of GO membranes in water environments^21,22^. Therefore, the reduced graphene oxide (rGO) membranes with lower oxygen functional groups can provide better performances in precise molecular sieving, due to their narrower nanochannels, lower swelling, and higher stable in water or harsh chemical conditions^23,24^. Unfortunately, water permeation through the stacked GO or rGO membranes is insufficient due to the strong capillary force and the narrow nanochannels^25^.

Broadening the interlayer spacings have been employed to modulate the water transport behaviors in GO membranes, generally via intercalation of soft polymers including cationic porphyrin^26^, polyacrylonitrile gel^27^, polyelectrolyte brushes^28^. However, incorporating polymer additives often cause very limited enhancement of water permeability, mainly owing to the intrinsic elastoplastic of the polymers and their uneven distribution on the GO surface^29,30^. In addition, the enlarged interlayer spacing will make a trade-off between permeability and selectivity, inducing significant rejection degradation^31,32^. It should be beneficial for tailoring the interlayer distance and improving membrane permeability if the intercalation materials are rigid and highly dispersed, but a practical intercalation strategy has remained elusive.

Herein, we report a general and facile colloidal synthesis to prepare ultrafine metal oxide/rGO nanocomposites for nanofiltration membranes. The synthesis is based on the utilization of oxygen functional groups on GO surface as preferential sites for fast heterogeneous nucleation, leading to the formation of sub-3 nm size, monodispersing as well as high-density loading of metal oxide nanoparticles on the rGO surface. The present synthesis is highly universal for anchoring various metal oxide nanoparticles, such as ZnO, CoO, CuO, MgO, Fe_2_O_3_, Nb_2_O_5_, CdO, La_2_O_3_, and MoO_3_ and metal sulfides such as ZnS, MoS_2_ nanoparticles. Moreover, the adhesion of these ultrafine nanoparticles could inhibit the wrinkling and restacking of the rGO nanosheet, forming highly stable colloidal solutions for low-cost solution processing of nanofiltration membranes. By functioning as rigid pillars, the nanoparticles not only increase the distance between the rGO sheets, but also create narrow tortuous paths among the 2D nanochannels for size-exclusion separation of dye molecules. The resulting membranes could realize high water permeability (225 L m^−2^ h^−1^ bar^−1^) and selectivity (up to 98%) of methyl blue, which places them among the most effective dye separation membranes reported to date. This study illustrates the utility of rigid nanoparticles as spacers for addressing the permeability-selectivity trade-off of GO-based membranes, providing insights into the design of next-generation nanofiltration membranes.

## Results

### Synthesis and characterization of ultrafine ZnO/rGO nanocomposites

**Fig. 1:**
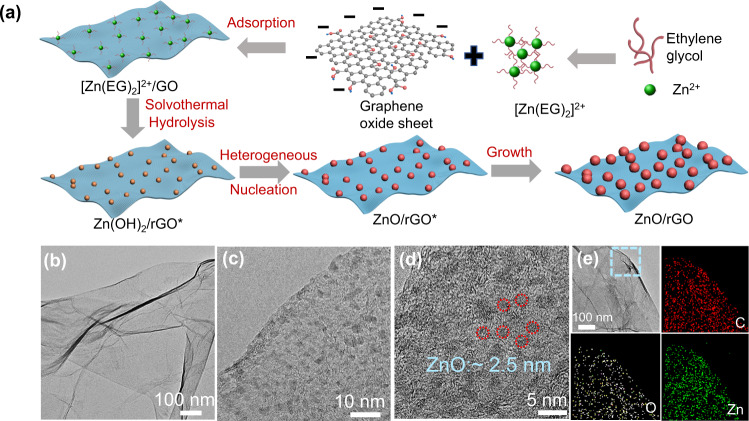
Synthesis and characterization of ultrafine ZnO/rGO nanocomposites. **a** Schematic illustration of the growth mechanism of ultrafine ZnO/rGO nanocomposites. The whole process is an adsorption-nucleation-growth process. Firstly, positively charged [Zn(EG)_2_]^2+^ complexes adsorbed to the negative charged GO, and then transformed into Zn(OH)_2_ and ZnO successively during solvothermal process. Finally, ultrasmall ZnO nanoparticles grow to larger ZnO nanoparticles on rGO surface. **b**–**d** TEM images of ultrafine ZnO/rGO nanocomposites at different magnifications. The red circles in d refer to ZnO nanoparticles. **e** TEM elemental mapping images of ultrafine ZnO/rGO nanocomposites.

Figure 1a illustrates a general heterogeneous nucleation and diffusion-controlled growth process of ultrafine metal oxide/rGO nanocomposites using ultrafine ZnO/rGO nanocomposites as an example. The GO is rich in oxygen functional groups, and it is supposed that these oxygen functional groups are responsible for the heterogeneous nucleation. More typically, in a mixed ethylene glycol (EG) solution of GO and zinc acetate dihydrates (Zn(Ac)_2_·2H_2_O), the presence of oxygen groups which contributes to an overall negatively charged surface of −40 mV will capture the positively charged [Zn(EG)_2_]^2+^ complexes through electrostatic interactions (Supplementary Fig. 1). Under solvothermal conditions, the adsorbed [Zn(EG)_2_]^2+^ complexes could be hydrolyzed to adsorbed Zn(OH)_2_, which is equivalent to the formation of preferential sites. At such sites, the effective surface energy is lower, thus diminishes the free energy barrier and facilitating heterogeneous nucleation. The as-formed nucleus will grow slowly via a diffusion-controlled growth process, leading to the formation of ultrafine and isolated island nanoparticles on the surface of rGO. The whole reaction process is schematically illustrated in Supplementary Fig. 2. Meanwhile, GO is reduced to rGO. As illustrated in Supplementary Fig. 3, GO is covalently functionalized with oxygen containing groups (hydroxyl, epoxide, carbonyl, etc.) on the basal plane and on the edges. These functional groups are unstable under high temperature. Thus, carbon monoxide (CO), carbon dioxide (CO_2_), and water (H_2_O) remove from GO surface during the solvothermal process, leading to the formation of rGO with lots of defects^33^.

**Fig. 2:**
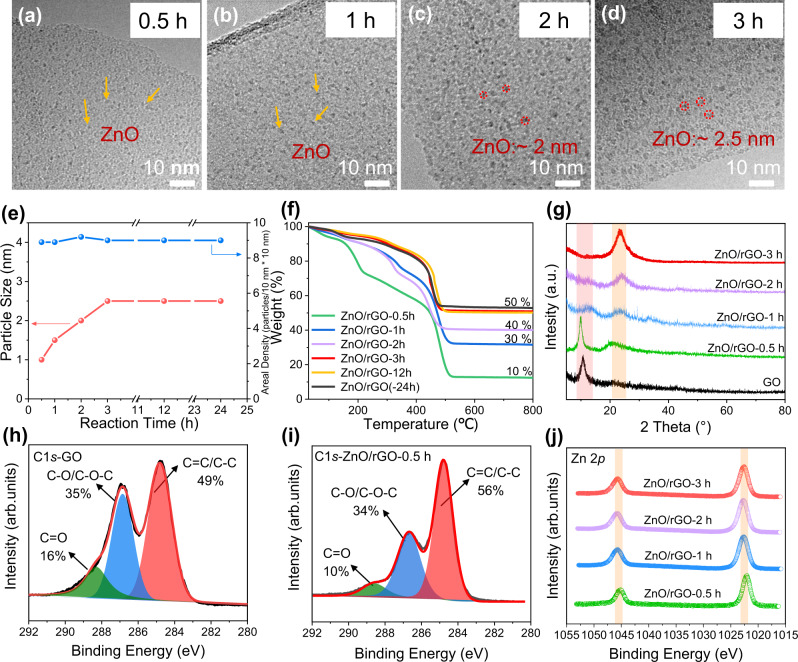
Mechanism study of the formation of ultrafine ZnO nanoparticles on GO surface. TEM images of (**a**) ZnO/rGO-0.5 h, (**b**) ZnO/rGO-1 h, (**c**) ZnO/rGO-2 h, and (**d**) ZnO/rGO-3 h. The yellow arrows and red circles refer to ZnO nanoparticles. **e** Graph of the average ZnO particle size and particle number density over time. **f** TGA curves of various samples. The residual weight of every sample after annealing in air at 800 °C refers to the ZnO contents in nanocomposites, which is given on the ride hand of every curve. **g** XRD spectra of various samples. The red and yellow shadings represent the XRD characteristic peak of GO and rGO, respectively. C1*s* XPS spectra of (**h**) GO, (**i**) ZnO/rGO-0.5 h. **j** Zn 2*p* XPS spectra of various samples. The yellow shadings represent the XPS characteristic peak of Zn 2*p*.

The TEM images reveal that the as-prepared ZnO/rGO nanocomposites has a very transparent laminated structure with less wrinkles and folding, which is similar to the pristine GO nanosheets (Fig. 1b). The high resolution TEM images (Fig. 1c, d) clearly demonstrate that the rGO surface is consisted of densely distributed ZnO nanoparticles with the size of ca. 2.5 nm. Besides, energy dispersive X-ray spectroscopy (EDS) elemental mappings (Fig. 1e) also confirm that ZnO nanoparticles are distributed homogeneously within the rGO nanosheets. The weight ratio of ZnO in the composite is determined to be close to the designed value 50 wt% (Fig. 2f), suggesting that all Zn precursors participate fully the hydrolysis reaction to ZnO.

### Mechanism study of the formation of ultrafine ZnO nanoparticles on GO surface

To understand the nucleation and growth of ZnO nanoparticles on rGO surface, the time-dependent experiments were conducted. At the initial solvothermal time of 0.5 h, the ZnO nanoparticles are already formed, but their size is only 1 nm corresponding to ~20 ZnO molecular clusters (Fig. 2a and Supplementary Fig. 4). With the time prolonging, the size gradually increases to ca. 2.5 nm at 3 h while remains unchanged until reaching to their final stage (Fig. 2b–e). It is interesting to note that the particle number density is almost the same of 9 per 10 × 10 nm for all samples (Fig. 2e and Supplementary Fig. 5), which clearly indicates that the nucleation occurs simultaneously at GO surface and no new nuclei form during the growth process. This result implies that the particle number density should be depended on the surface characteristics of GO, more especially the number of oxygen functional groups.

To verify the structure and chemical state of the nanocomposites produced at the first few hours of solvothermal synthesis, the thermogravimetric (TG), X-ray photoelectron (XPS), and X-ray diffraction (XRD) characterizations were conducted. The weight ratio of ZnO in composites from TG results (Fig. 2f) has a similar increasing tendency with the size of ZnO nanoparticles. At the initial 0.5 h, the weight ratio of ZnO nanoclusters is only 10 wt%, and then gradually increases to the designed value of 50 wt% at 3 h. It is noted that the ZnO/rGO-0.5 h has a significant weight loss at temperature of 160–220 °C in TG curve, which should be due to deoxygenation of incompletely reduced GO. This could be well confirmed by XRD pattern (Fig. 2g), in which the ZnO/rGO-0.5 h exhibit two shoulder peaks centered at 11^o^ and 23^o^, responding to the characteristic of GO and rGO^34,35^, respectively. And after the further solvothermal reduction of 1 h, the GO peak at 11^o^ disappears mostly and the peak at 23^o^ gets dominant, which indicates the coexistence of rGO and GO in ZnO/rGO-1 h. While the time increased to 2 h, only a characteristic peak of rGO at 23^o^ could be discerned, verifying that the reduction of GO could be accomplished at the initial 2 h under solvothermal condition. And no obvious characteristic peaks could be discerned for the ZnO crystals, further confirming their ultrafine size. In addition, XPS patterns (Fig. 2h, i and Supplementary Figs. 6, 7) were carried out. The high resolution C1*s* spectrum of GO are deconvoluted into three major components, including C=C/C-C (284.8 eV), C-O/C-O-C (286.1–287.1 eV) and C=O/O-C=O (288.2–289.3 eV)^36^. C=C/C-C is contributed to *sp*^2^ and *sp*^3^ hybridized carbon atoms disposed in two-dimensional GO layer, C-O/C-O-C is result from hydroxyl and epoxy, and C=O/O-C=O exist in carbonyl and carboxyl^37^. Upon 0.5 h solvothermal reaction, the fraction of the C-C/C=C groups in C1*s* spectra show a significant increase from 49% to 56%, while the intensities of C=O and C-O/C-O-C groups drop, which can be attributed to the breakup of oxygen functional groups (Fig. 2g). After reacting 3 h, the intensity of C-C/C=C groups in Supplementary Figs. 6, 7 further increases, and the proportion of C=O groups remains drop, suggesting almost reduction of GO in ZnO/rGO-3 h. During the deoxygenated process, CO_2_, CO, and H_2_O were produced, leading to decrease of O ratio and increase of C ratio. It should be noted that the Zn 2*p* spectra (Fig. 2j) for all samples are very similar with two obvious bands located at 1045.3 eV and 1022.3 eV, corresponding to the Zn^2+^ 2*p*_1/2_ and Zn^2+^ 2*p*_3/2_, respectively^38^. All these results indicate that the reduction of GO and the growth of ZnO on rGO nanosheets could be accomplished at the initial 3 h under solvothermal conditions.

**Fig. 3:**
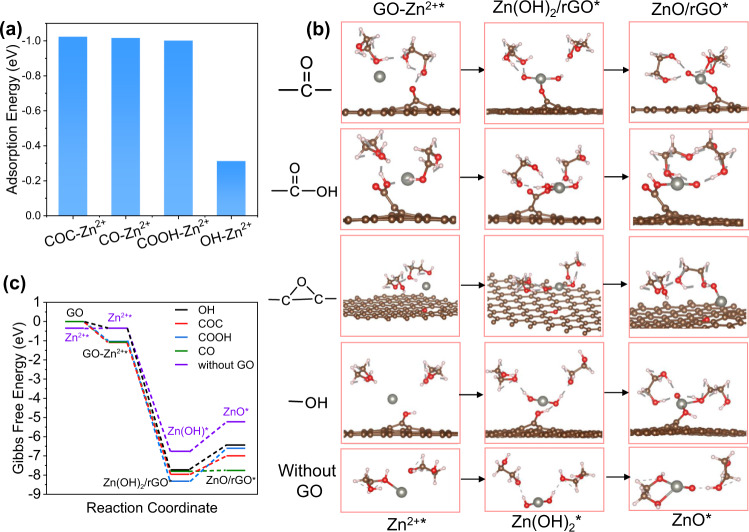
DFT calculations of the formation of ultrafine ZnO nanoparticles on GO surface. **a** Adsorption Energy of Zn^2+^ on oxygen functional groups. **b** The optimized adsorption conformations of intermediate species on GO surface oxygen functional groups and in the absence of GO in EG. Zn, C, O and H atoms are represented by gray, brown, oxygen, and pink balls, respectively. **c** Energy profiles for the formation of ZnO nanoparticles on GO surface functional groups and in the absence of GO in EG.

The key to the synthesis of ultrafine ZnO nanoparticles should be the oxygen functional groups on GO surface, which have binding ability toward Zn^2+^ through electrostatic attractions, and consequently trigger the heterogenous nucleation. According to the elemental analysis, GO used in this work has a C/O ratio of ca.1.8. And XPS spectrum in Fig. 2h reveals that it is consisted of majorly epoxide and carbonyl functional groups on the basal plane, and hydroxyl and carboxyl groups on its edges^39^. To get insights into the binding abilities of these oxygen functional groups with Zn^2+^ in EG, we perform density functional theory (DFT) calculation and disclose that the order of adsorption stability toward Zn^2+^ is as follows: epoxy(-COC) > carbonyl (-CO) > carboxyl (-COOH) » hydroxyl (-OH) (Fig. 3a). Furthermore, the reaction barriers of the hydrolysis of adsorbed Zn^2+^ and then its transformation to ZnO on GO surface and without GO are analyzed. The optimized structures of intermediates and their Gibbs free profiles are displayed in Fig. 3b, c and Supplementary Table 1. The overall Gibbs free energy changes on oxygen functional groups (-OH, -COC, -COOH, and -CO) in EG reaction system are −6.433 eV, −6.999 eV, −6.608 eV, and −7.759 eV, respectively, which are all more negative than that of without GO (−4.783 eV). The lower Gibbs Energy changes lead to smaller reaction barrier. The last step of Zn(OH)_2_/rGO*→ZnO/rGO* exhibits positive Gibbs energy change (ΔG), which infers this step is the thermodynamic-limiting step in the whole process. From the results of Gibbs energy changes, it can be concluded that the total transformation process of Zn^2+^ to ZnO is a spontaneous process and thermodynamically more favorable on GO surface. The carbonyl is suggested as the most active site for nucleation and followed by epoxy and carboxyl.

The controlled nucleation is triggered by the oxygen functional groups on GO surface due to the lower free energy barrier, while the consequent growth of ZnO nanoparticles should be a diffusion-controlled process that is limited by the rate of incorporation of adatoms into ZnO growth centers. Herein, the growth of ZnO nanoparticles on the rGO surface was studied through a series of control experiment by changing the reaction temperature. As shown in Supplementary Fig. 8a, the ZnO/rGO nanocomposites prepared at temperature of 90 °C show ultrasmall ZnO nanoparticles of ca. 1.3 nm. Such low nucleation temperature should be due to the lowering heterogenous nucleation barrier, while the small size should be caused by the limited diffusion at low temperature. With increasing the reaction temperature to 180 °C, the average size of ZnO nanoparticle increases gradually, but the particle number density keeps almost unchanged on the rGO surface (Supplementary Figs. 8–10). In addition, the concentration of precursor Zn (Ac)_2_·2H_2_O was also changed to adjust the loading contents of ZnO in ZnO/rGO nanocomposites. At a low precursor concentration, the TG curve reveals that the content of ZnO is 30 wt%, being the same with the designed value (Supplementary Fig. 10b). However, the resulting nanocomposites (Supplementary Fig. 8d) show an almost transparent feature without the visible nanoparticles on the rGO surface, which should be due to the formation of ultrasmall ZnO clusters (<1 nm) that are hardly displayed by the TEM images. While the ZnO loading is larger than 50%, the nanocomposites show almost similar morphologies of ZnO nanoparticles (Supplementary Figs. 8e, f, 10b).

Furthermore, the synthesis of pure ZnO particles without GO was conducted under the same solvothermal conditions. The obtained ZnO product shows spherical aggregates with size of ca. 600 nm (Supplementary Fig. 11), which should be due to the homogenous nucleation continued by the growth of primary nanoparticles and thereafter the spherical aggregation. In addition, the solvent is also critical for the formation uniform ZnO nanoparticles on the rGO surface. EG is selected as the solvent due to its strong chelating and reduction ability^40^. While changing solvent from EG to deionized water and ethanol (Supplementary Figs. 12, 13), large and aggregated particles are formed on the wrinkled rGO surface.

### General synthesis of ultrafine metal oxide/rGO nanocomposites

**Fig. 4:**
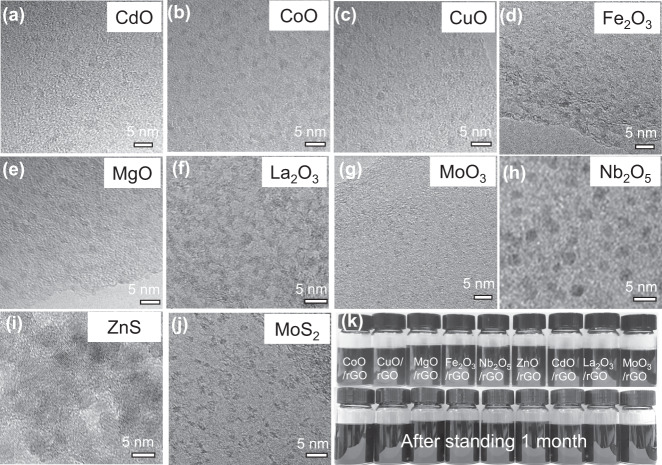
Characterization of other ultrafine metal oxide/rGO nanocomposites. TEM images of (**a**) CdO/rGO, (**b**) CoO/rGO, (**c**) CuO/rGO, (**d**) Fe_2_O_3_/rGO, (**e**) MgO/rGO, (**f**) La_2_O_3_/rGO, (**g**) MoO_3_/rGO, (**h**) Nb_2_O_5_/rGO, (**i**) ZnS/rGO, and (**j**) MoS_2_/rGO nanocomposites, respectively. **k** Colloidal solutions of various metal oxide/rGO after and before standing 1 month.

Based on the above discussion, the oxygen functional groups-induced heterogenous nucleation should work as a general strategy for the synthesis of other metal oxide/rGO nanocomposites. Herein, a family of nanocomposites with ultrafine metal oxide nanoparticles grown on rGO surface are successfully prepared, including CdO/rGO, CoO/rGO, CuO/rGO, Fe_2_O_3_/rGO, MgO/rGO, La_2_O_3_/rGO, MoO_3_/rGO, and Nb_2_O_5_/rGO. As shown in Fig. 4a–h and Supplementary Figs. 14, 15, TEM images verify that all nanocomposites consist of monodispersed ultrafine metal oxide nanoparticles with the size less than 3 nm. Moreover, metal sulfide nanoparticles/rGO nanocomposites, such as ZnS/rGO and MoS_2_/rGO could also be prepared by the present strategy through adding the thiourea in the precursor solution, as shown in Fig. 4i, j and Supplementary Fig. 16.

The synthetic procedures reported in this work are highly reproducible and readily applicable for the large-scale synthesis of ultrafine metal oxide/rGO nanocomposites. And more importantly, due to the effective inhibition of wrinkling of the rGO sheets, the as-prepared nanocomposites are highly dispersed into EG, forming a homogenous colloidal suspension of ca. 4 mg/ml without obvious sedimentation even after 1 month (Fig. 4k). Furthermore, the as-prepared nanocomposites could be also dispersed in the organic solvent such as N, N-dimethylformamide (DMF), isopropanol or N-methylpyrrolidone (NMP), which are desirable for wet processing to enable a homogenous arrangement of nanocomposites as filler, coatings, or thin films.

### Preparation and characterization of ZnO/rGO membranes

**Fig. 5:**
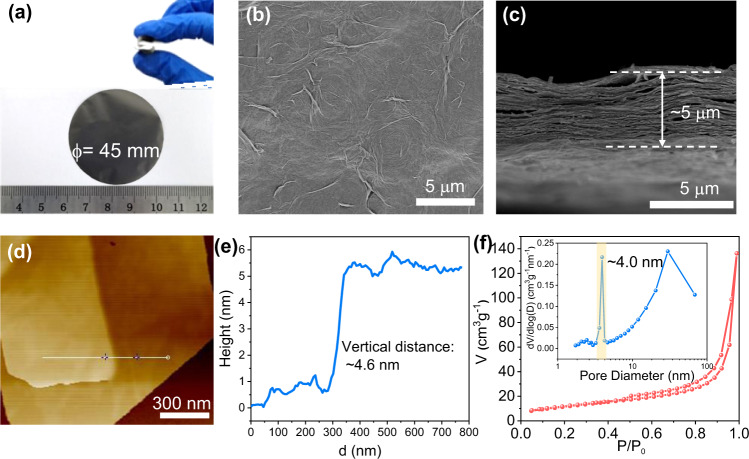
Characterizations of ZnO/rGO membranes. **a** Digital photographs of the ZnO/rGO membranes, (**b**) top-view SEM image, and (**c**) cross-section SEM image of ZnO/rGO membranes. **d**, **e** AFM images of ZnO/rGO membranes. **f** N_2_ adsorption-desorption isotherm and corresponding pore size distribution (inset) of ZnO/rGO membranes.

The free-standing ZnO/rGO nanocomposite membranes were fabricated by a simple vacuum filtration of homogenous colloidal suspensions. After that, being washed with ethanol and dried at 60 °C, the ZnO/rGO membrane with 45 mm diameter is obtained (Fig. 5a). This membrane can randomly bend 360°, suggesting its excellent flexibility and durability. SEM images (Fig. 5b) indicate that the resulting membrane is continuous and free of macropores or surface defects. And the cross-section image (Fig. 5c) demonstrates a layered structure resembling that of nacre, therefore exhibiting good mechanical strength. Such layered structure should be due to the continuous suction force from vacuum pump, which can move the solvent rapidly that can overcome the agglomeration of nanocomposites and form the laminated membranes. In addition, the surfaces of rGO nanosheets parallel to the basic membranes are more kinetically favorable than that of the nanosheets perpendicular to the membranes^41–43^.

The distance between ZnO/rGO bilayer is ∼4.6 nm determined by AFM images (Fig. 5d, e). By subtracting the rGO monolayer (ca. 0.34–0.4 nm)^23^, the 2D nanochannel spacing of ZnO/rGO membrane is estimated at ∼4.2 nm, which is five times that of GO membranes (Supplementary Fig. 20 and Fig. 2g). In addition, the roughness of GO and ZnO/rGO membranes are given in Supplementary Table 2. Compared with GO membranes, ZnO/rGO membranes show obviously increasing roughness degree, which is due to the insertion of rigid ZnO nanoparticles. Furthermore, the interlayer spacings between nanosheets as pores are evaluated by nitrogen adsorption-desorption measurement. As shown in Fig. 5f, ZnO/rGO membrane displays a Type IV sorption isotherm with Type H3 hysteresis^44^, responding to the slit-shaped pores between nanosheets. The resulting BJH pore size distribution reveals that the average pore size is at ∼4.0 nm, which is consistent with AFM results.

### Nanofiltration performance of ZnO/rGO membranes

**Fig. 6:**
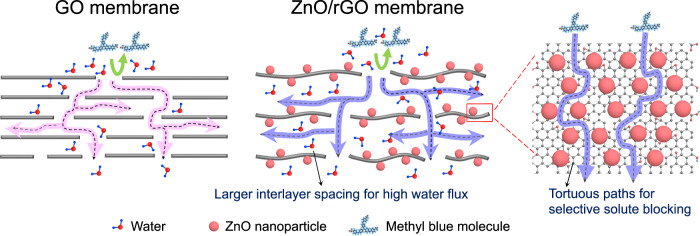
Schematic illustration of separation mechanism and water transport through GO membranes and ZnO/rGO membranes. Insertion of rigid ZnO nanoparticles between rGO layers increase both the interlayer spacings and in-plane obstructor of rGO layers, leading to higher water permeance and dye molecule selectivity. The green arrows represent the rejection of MB (methyl blue) molecules, the pink arrow represents flow path of water in GO membranes, and the purple arrows represent flow path of water in ZnO/rGO membranes.

GO-based membranes are great promising for advanced nanofiltration in water treatments, however, their narrow interlayer channels generally limit the water flux and the separation of larger organic molecules^45^. Generally, the separation mechanism of GO membranes is governed primarily by the interlayer spacing between the nanosheets and the length or tortuosity of the transport pathways^46^. By functioning as pillars, the metal oxide nanoparticles remarkably increase both vertical interlayer spacing and lateral tortuous paths of the rGO membranes, offering the great possibility for the membranes concurrently with high water permeance and high rejections (as illustrated in Fig. 6).

To evaluate the separation performance, we fabricated the ZnO/rGO membranes supported by nylon substrates and used them for dye permeation tests. As shown in Supplementary Fig. 21, ZnO/rGO membranes with the layer-by-layer structure are tightly coated on a porous nylon 66-0.22 μm substrate via vacuum filtration with 1 bar vacuum. The surface wettability of ZnO/rGO membranes is determined to be ca. 46^o^ by the water contact angle (Supplementary Fig. 22), indicating excellent hydrophilic properties. Due to the supporting of porous nylon, the large-area ZnO/rGO membranes with the diameter of ca. 75 mm can also be manufactured (Supplementary Fig. 23). In addition, the loading mass of ZnO/rGO on nylon membranes can be changed from 0.14 to 0.58 mg cm^−2^ by varying the volume of colloidal dispersion during the vacuum filtration. The average thicknesses of the ZnO/rGO membranes are in the range of nanometers (220 nm) to micrometers (1.1 μm) estimated by the SEM observation (Supplementary Fig. 23).

**Fig. 7:**
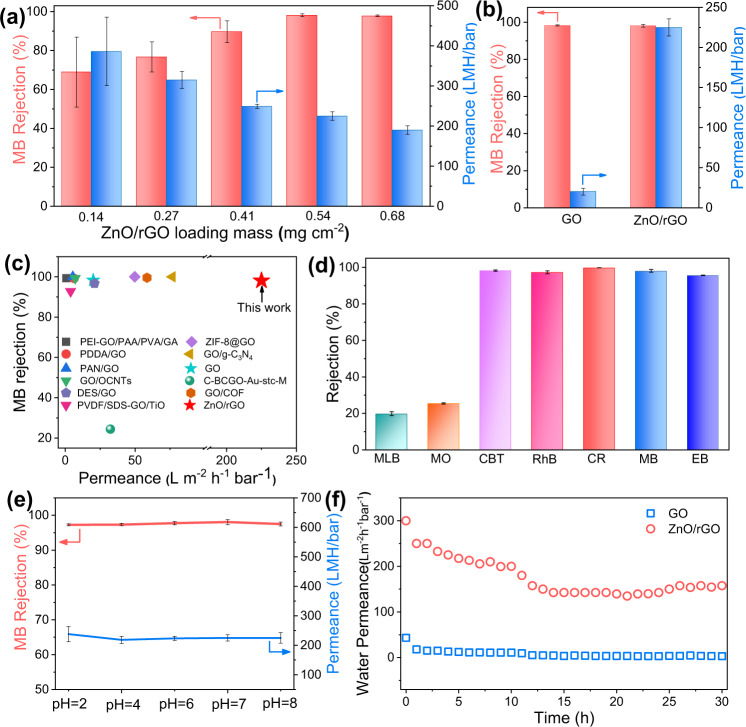
Nanofiltration performance of ZnO/rGO and GO membranes. **a** Water permeance and MB (methyl blue) rejection for ZnO/rGO membranes with different loading mass. The blue and red arrows represent data corresponding to water permeance and MB rejection, respectively. Error bars represent standard deviations from measurements of three different samples. **b** Water permeance and MB rejection for ZnO/rGO membranes and GO membranes. Error bars represent standard deviations from measurements of three different samples. **c** Comparation of water permeance and MB rejection of reported GO-based membranes. **d** Separation performance of ZnO/rGO for dye molecules of varying molecular weight: Evans Blue (EB, M_W_ = 960), Methyl Blue (MB, M_W_ = 799.8), Congo Red (CR, M_W_ = 696), Rhodamine B (RhB, M_W_ = 479), Chrome Black T (CBT, M_W_ = 461), Methyl Orange (MO, M_W_ = 327) and Methylene Blue (MLB, M_W_ = 319.85). Error bars represent standard deviations from measurements of three different samples. **e** Water permeance and MB rejection for ZnO/rGO membranes at different pH values. Error bars represent standard deviations from measurements of three different samples. **f** Pure water permeance of ZnO/rGO membranes and GO membranes over a 30 h operating period.

Due to the variations in transport distance, the loading mass of nanosheets has a critical effect on the water flux and the rejection rate of methyl blue (MB), as shown in Fig. 7a. With the increase of the ZnO/rGO loading mass, the rejection rate increased from 69% to 98.1%, while water flux decreases from 390 to 190 L m^−2^ h^−1^ bar^−1^. Therefore, we select the optimized loading mass of ca. 0.54 mg cm^−2^ for further performance evaluation, which achieve simultaneously an extraordinary high water flux of 225 L m^−2^ h^−1^ bar^−1^ and MB rejection of 98.1 %. To highlight the advanced performance of ZnO/rGO membranes, the pure GO membranes at the same mass loadings are prepared, which exhibits only 20 L m^−2^ h^−1^ bar^−1^ water permeance as given in Fig. 7b. Moreover, the MB rejections of other reported GO-based membranes are compared in Fig. 7c and Supplementary Table 2. Except the exciting rejection rate, the ZnO/rGO membranes exhibit much higher water permeance than those of the GO-based membranes reported so far (generally less than 80 L m^−2^ h^−1^ bar^−1^)^47–57^.

Molecular selectivity is one of crucial parameters for the nanofiltration. Here we select a range of dye molecules with different weights (319.85 ∼ 960 Da) including evans blue (EB), congo red (CR), Rhodamine B (RhB), chrome black T (CBT), methyl orange (MO), and methylene blue (MLB) comparison. Their chemical structures, surface charge, and molecular sizes are given in Supplementary Table 3. As shown in Fig. 7d, the ZnO/rGO membranes still exhibit remarkable high rejections of >95% for EB, CR, RhB, and CBT at high water permeances of ∼220 L m^−2^ h^−1^ bar^−1^, regardless of solute charge properties. However, in the case of MO and MLB with small molecular size of 1.13 × 0.42 nm and 1.25 × 0.51 nm, the ZnO/rGO membranes show only 27% and 20% rejection as a result of ineffective sieving effects for smaller molecules. Furthermore, the separation of a mixed solution of methyl blue/methyl orange (MB/MO) was carried out. As shown in Supplementary Fig. 26, the ZnO/rGO membranes remain MB rejection of 98% and MO rejection of 25%, being similar to the results of single component solution filtration test.

The membrane potential for extreme pH nanofiltration is key to practical applications. Here, we carried out the performance of ZnO/rGO membranes at MB feed pH values from 2 to 8, as shown in Fig. 7e. Under a board pH condition, ZnO/rGO membranes still have an extraordinary high water flux of ∼225 L m^−2^ h^−1^ bar^−1^ and MB rejection of ∼98%. Furthermore, the mechanical stability of nanofiltration membranes is also very critical in practical applications. The long-term stability of ZnO/rGO and GO membranes of pure water were also conducted over a duration of 30 h. As shown in Fig. 7f, the GO membranes deliver an initial water flux of 20 L m^−2^ h^−1^ bar^−1^ and then decline rapidly to 3 L m^−2^ h^−1^ bar^−1^, due to the compression of loosely overlapped GO nanosheets under pressure. In contrast, the rigid ZnO nanoparticles could serve as rigid “pillars” to create permanent high-speed waterways along the surface of rGO nanosheets, achieving a high steady-state flux value of 160 L m^−2^ h^−1^ bar^−1^ after steadily 30 h working.

## Discussion

The separation mechanism of ZnO/rGO membranes for dye molecules should be dominated by size-exclusion selectivity, based on the counteracting roles of enlarging interlayer spacing and in-plane steric effects both caused by high-density loading of ultrafine ZnO nanoparticles. Water molecules could easily pass through the spaces between the pillars, while large-molecular dyes are selectively blocked based on their size and shape. The ZnO/rGO membranes thus address the critical trade-off between water flux and dye rejection in contrast to conventional nanofiltration membranes. Furthermore, the membrane microstructure could be adjusted by using different nanoparticle sizes and loadings, allowing us to control both the height and width of the paths between the pillars, thereby offering the great possibility towards on-demand nanofiltration applications.

In conclusion, we demonstrate a general and facile method to synthesize ultrafine metal oxide/rGO nanocomposites through heterogenous nucleation and growth process for nanofiltration applications. The key to the present synthesis is employing the oxygen functional groups on GO, which have binding ability toward Zn^2+^ through electrostatic attractions, and sequential triggers the heterogenous nucleation. Another important merit of the present synthesis is the effective inhibition of wrinkling and stacking of the resulting rGO sheets via the uniform adhesion of these ultrafine metal oxide nanoparticles. The adhesion of these metal oxide nanoparticles not only leads to physical separation of the rGO sheets, but also forms stable dispersions for wet processing of the membranes. Water molecules could easily pass through the narrow spaces between the nanoparticles, while large-molecular dyes are selectively rejected based on their size and shape. Moreover, the membrane microstructure can be turned vertically and laterally by using different sizes and loadings of metal oxide nanoparticles, extending their great potentials for on-demand applications such as water treatment, solvent dehydration, and organics sieving.

## Methods

### Chemicals

All chemicals were of analytic grade. Zinc Acetate Dihydrate was purchased from General-Reagent. Lanthanum (III) acetate hydrate (La(Ac)_3_·6H_2_O), ammonium molybdate (VI) tetrahydrate (Mo_7_O_24_(NH_4_)_6_), chromium (II) acetate dihydrate (Cd(Ac)_2_·2H_2_O), cobalt (II) acetate tetrahydrate (Co(Ac)_2_·4H_2_O), cupric (II) acetate monohydrate (Cu(Ac)_2_·H_2_O), ferric (II) acetate tetrahydrate (Fe(Ac)_2_·4H_2_O), magnesium (II) acetate (Mg(Ac)_2_), and ethylene glycol (EG) were purchased from Macklin. Niobium (V) pentachloride (NbCl_5_) was purchased from Adamas-beta. All chemicals were used without further purification.

### Synthesis of ultrafine ZnO/rGO nanocomposites

The GO suspension was provided by The Institute of Coal Chemistry, Chinese Academy of Sciences and prepared by a modified Hummers method from natural graphite^58^. The as-prepared GO nanosheets were frozen-dried under −90 °C and then dispersed into EG under ultrasonic and magnetic stirring to form a homogenous solution of 5 mg/ml. And 0.27 g zinc acetate dihydrate (Zn(Ac)_2_·2H_2_O) was dissolved in a 30 mL EG solution with ultrasound for 1 h to form a transparent solution. Then, the above Zn(Ac)_2_·2H_2_O/EG solution was added to 25 mL 5 mg/ml GO/EG solution dropwise. After continuous magnetic stirring for about 2 h, the resultant solution 55 mL Zn (Ac)_2_-GO/EG solution was transferred into a 100 mL Teflon-lined stainless-steel autoclave and heated at 180 °C for 24 h. After cooling down to room temperature naturally, the ultrafine ZnO/rGO nanocomposites were obtained. The designed ZnO content in the composite is 50%.

In a control experiment, the solvothermal time was changed to 0.5 h, 1 h, 2 h, 3 h, 8 h, and 12 h, ZnO/rGO-1 h, ZnO/rGO-2 h, ZnO/rGO-3 h, ZnO/rGO-8 h, and ZnO/rGO-12 h were obtained, respectively. The samples prepared at different temperatures of 90 °C, 120 °C, and 150 °C were denoted as ZnO/rGO-90 °C, ZnO/rGO-120 °C, and ZnO/rGO-150 °C, respectively. The samples prepared with different Zn(Ac)_2_·2H_2_O weight of 0.12 g and 0.63 g were denoted as ZnO/rGO-30% and ZnO/rGO-70%, respectively. The samples synthesized in different solvents of deionized water and ethanol were denoted as ZnO/rGO-H_2_O and ZnO/rGO-EtOH. Pure ZnO was synthesized without adding GO/EG suspension in preparation.

### Synthesis of other ultrafine metal oxide/rGO nanocomposites

All ultrafine metal oxide/rGO nanocomposites (CdO/rGO, CoO/rGO, CuO/rGO, Fe_2_O_3_/rGO, MgO/rGO, La_2_O_3_/rGO, MoO_3_/rGO, and Nb_2_O_5_/rGO) were synthesized using the same process as that of ultrafine ZnO/rGO nanocomposites, except that Zn(Ac)_2_·2H_2_O was replaced by other metal salts (Cd(Ac)_2_·2H_2_O, Co(Ac)_2_·4H_2_O, Cu(Ac)_2_·H_2_O, Fe(Ac)_2_·4H_2_O, Mg(Ac)_2_, La(Ac)_3_·6H_2_O, Mo_7_O_24_(NH_4_)_6_·4H_2_O, and NbCl_5_). The designed contents of all metal oxide in nanocomposites are 50%.

### Synthesis of ultrafine metal sulfide/rGO nanocomposites

The ultrafine ZnS/rGO and MoS_2_/rGO nanocomposites were prepared in the same way of ZnO/rGO and MoO_3_/rGO nanocomposites, respectively, through adding the thiourea in the precursor solution. The designed contents of all metal sulfide in nanocomposites are 50%.

### Preparation of ZnO/rGO free-standing membranes

As-prepared ultrafine ZnO/rGO nanocomposites were dispersed into EG to form a homogenous solution ca. 0.34 mg/ml. And then, the solution was vacuum filtered with polyvinylidene difluoride (PVDF) membranes. The wet ZnO/rGO nanocomposites with PVDF membranes were fabricated and washed with ethanol several times. After being dried in 60 °C for 12 h, the ZnO/rGO membranes could be peeled off as a free-standing membrane.

### Materials characterizations

The morphologies and structures of nanocomposites were observed by using transmission electron microscopy (TEM, JEM-2100), scanning electron microscopy (SEM, Nova NanoSEM 450), and atomic force microscopy (AFM, MFP-3D). The thermogravimetric analysis (TGA 8000) was carried out at an air flow of 30 mL min^−1^ with heating from 30 °C to 800 °C at a rate of 10 °C min^−1^. The X-ray diffraction (XRD) patterns were obtained by using Cu Kα radiation (*λ*=1.5406 Å) on a Rigaku D/Mac 2550 diffractometer. The X-ray photoelectron spectroscopy (XPS) was carried out using ESCALAB 250Xi. Zeta potential pattern was obtained by using a Zetasizernano. The elemental composition was obtained from the CHN elemental analysis (Elementar Vario EL; Thermo Fisher). The N_2_ adsorption-desorption isotherm was carried out by ASAP2460, while the pore size was determined by Barrett–Joyner–Halenda (BJH) theory.

### Density Functional Theory calculations

All the calculations were carried out by using the Perdew-Burke-Ernzerhof (PBE) function based on density functional theory (DFT) within the Vienna Ab Initio Simulation Package (VASP). To describe the electron−ion interactions, the cutoff energy of the plane wave was set as 400 eV. All atomic structures were allowed to relax until the maximum force was smaller than 0.02 eV/Å with the energy convergence criterion of 10^−5^ eV. And the 25 Å was set as the thickness of vacuum layer to avoid periodic effects between mirror images. The graphene sheets containing defects were simulated by the 8 × 8 × 1 supercells of repeated model. Integration of the Brillouin zone was performed by 1 × 1 × 1 k-point grids based on Gamma Scheme to obtain the optimized configuration and total energy of the system. What’s more, Bader charge difference analysis was adopted to describe the charge transfer within the system.

The adsorption energy (*E*_ads_) was calculated according to the following equation:

1 $${E}_{{{{{{\rm{ads}}}}}}}={E}_{{{{{{\rm{adsorbate}}}}}},{{{{{\rm{sub}}}}}}}-{E}_{{{{{{\rm{sub}}}}}}}-{E}_{{{{{{\rm{adsorbate}}}}}}}$$

Where E_adsorbate, sub_ represents the total energy of the adsorbate on the substrate, E_sub_ represents the energy of the adsorbate, and E_adsorbate_ represents the energy of the adsorbate.

The Gibbs free energy difference (Δ*G*) of reaction pathways was calculated according to the following equation:

2 $${\Delta }G={\Delta }H+{\Delta }{E}_{{{{{{\rm{zpe}}}}}}}-T\Delta S$$

Where Δ*H*, Δ*E*_zpe_, and Δ*S* are the difference of enthalpy, the zero-point energy, and the entropy of each elementary reaction, respectively. Set *T* to the appropriate temperature in each calculation step. In addition, Δ*E*_*zpe*_ is attained through vibration frequency analysis.

### Evaluation of nanofiltration performance

ZnO/rGO and GO colloidal solutions were loaded on nylon substrates by a simple vacuum filtration. For vacuum filtration process, nylon membrane was firstly wetted by water, and then it was fixed onto the sand core of the suction filter equipment by a big holder. After that, highly dispersed ZnO/rGO (or GO) solution was poured onto the surface of nylon membrane with a loading mass of 0.54 mg cm^−2^ by controlling the volume of colloidal solutions (0.34 mg/ml) at 20 ml. At the same time, a vacuum pump was connected to make the liquid penetrating through the porous nylon membrane until no visible liquid on the surface of the membrane. Each membrane was analyzed in triplicates under the same conditions. The nanofiltration performance was determined using dead-end filtration of various dye solutions (25 ppm). The pH of the solution was adjusted by 0.01 mol/L HCL and KOH solution. The feed and permeate concentrations were analyzed by ultraviolet-visible (UV-Vis) spectroscopy, which was measured at the maximal absorption wavelength of the five organic dyes.

Filtration characteristics, including water permeability (*J*, L m^−2^ h ^−1^ bar^−1^) and dye rejection (*R*, %), were calculated by the following equations:

3 $$J=\frac{{J}_{W}}{\Delta P}=\frac{V}{A{{\times }}t{{\times }}\Delta P}$$

Where V is the volume of permeate (*L*), A is effective membrane area (4 × 10^−4^ m^2^,), t is the permeation time (*h*), and ΔP is the transmembrane pressure (1 bar).

4 $${R}_{( \% )}=\frac{{C}_{f}-{C}_{P}}{{C}_{f}}{{\times }}100$$

Where *C*_f_ and *C*_p_ are the concentrations of markers in the permeate and retentate solutions.

## Supplementary information

Supplementary Information

## Data Availability

The data that support findings from this study are available from the corresponding author on request.
