# Supplementary material for: General synthesis of ultrafine metal oxide/reduced graphene oxide nanocomposites for ultrahigh-flux nanofiltration membrane
Source: Nat Commun. 2022 Jan 25;13:471. doi: 10.1038/s41467-022-28180-4 (PMC8789770; doi:10.1038/s41467-022-28180-4)
Supplement: Supplementary file 1 — Supplementary Information [file 41467_2022_28180_MOESM1_ESM.pdf]

Supplementary Information:

General Synthesis of Ultrafine Metal Oxide/Reduced Graphene Oxide Nanocomposites for Ultrahigh-Flux Nanofiltration Membrane

Wanyu Zhang[a], Hai Xu[a], Fei Xie[a], Xiaohua Ma[a], Bo Niu[a*], Mingqi Chen[a], Hongyu Zhang[a], Yayun Zhang[a], Donghui Long[a*]

[a]State Key Laboratory of Chemical Engineering, East China University of Science and Technology, Shanghai 200237, China

These authors jointly supervised this work: Bo Niu, Donghui Long.

*Email: niubo@ecust.edu.cn; longdh@ mail.ecust.edu.cn

Figures and Tables:

Supplementary Figure 1. Zeta potentials of GO, Zn $(Ac)_2 \cdot 2H_2O$ and GO-Zn $(Ac)_2 \cdot 2H_2O$ mixture.

Supplementary Figure 2. The reaction process of the formation of ZnO/rGO nanocomposites.

Supplementary Figure 3. Schematic illustration of the reduction of GO.

Supplementary Figure 4. Illustration of one ZnO cell (Zn white, O red; highlighted atoms are inside unit cell).

Supplementary Figure 5**.** TEM images of various samples synthesized at (a) 0.5 h, (b) 1 h, (c) 2 h, (d) 3 h, (e) 12 h and (f) 24 h.

Supplementary Figure 6. High-resolution C 1s XPS spectrum curves of ZnO/rGO-3h.

Supplementary Figure 7. High-resolution C 1s XPS spectrum curves of ZnO/rGO.

Supplementary Figure 8. HR-TEM images of (a) ZnO/rGO-90°C, (b) ZnO/rGO-120°C, (c) ZnO/rGO-150°C, (d) ZnO/rGO-30%, (e) ZnO/rGO and (e) ZnO/rGO-70%.

Supplementary Figure 9. Graph of the average particle size and particle number density over temperature.

Supplementary Figure 10. Thermogravimetric analysis (TGA) curves of samples prepared (a) at various temperature and (b) by various precursor concentration.

Supplementary Figure 11. (a-b) TEM images, (c) XRD pattern, and SEM image of ZnO.

Supplementary Figure 12. (a-b) TEM images of ZnO/rGO-H_2O.

Supplementary Figure 13. (a-b) TEM images of ZnO/rGO-EtOH.

Supplementary Figure 14. TEM images of (a) CdO/rGO, (b) CoO/rGO, (c) CuO/rGO, (d) Fe_2O_3/rGO nanocomposites at different magnifications.

Supplementary Figure 15. TEM images of (a) MgO/rGO, (b) La_2O_3/rGO, (c) MoO_3/rGO, (d) Nb_2O_5/rGO nanocomposites at different magnifications.

Supplementary Figure 16. TEM images of (a) ZnS/rGO and (b) MoS_2/rGO nanocomposites at different magnifications.

Supplementary Figure 17. (a) Cd 3d XPS pattern of CdO/rGO. (b) Co 2p XPS pattern of CoO/rGO. (c) Cu 2p XPS pattern of CuO/rGO. (d) Fe 2p XPS pattern of Fe_2O_3/rGO. (e) Mg 1s and (f) Mg 2p XPS patterns of MgO/rGO. (g) La 3d XPS pattern of La_2O_3/rGO. (h) Mo 3d XPS pattern of MoO_3/rGO. (i) Nb 3d XPS pattern of Nb_2O_5/rGO.

Supplementary Figure 18. Thermogravimetric analysis (TGA) curves of CdO/rGO, CoO/rGO, CuO/rGO, Fe_2O_3/rGO, La_2O_3/rGO, MgO/rGO, MoO_3/rGO, ZnO/rGO and Nb_2O_5/rGO under air atmosphere.

Supplementary Figure 19. Illustration of the preparation of ZnO/rGO membranes through vacuum filtration

Supplementary Figure 20. (a-b) AFM images of GO.

Supplementary Figure 21. (a) Digital photograph, (b) top-view SEM image and (c) cross-section SEM image of ZnO/rGO/nylon membranes.

Supplementary Figure 22. Contact angle of water on (a) GO/Nylon and (b) ZnO/rGO/Nylon.

Supplementary Figure 23. Example of a large-area ZnO/rGO/nylon membrane fabricated by vacuum filtration.

Supplementary Figure 24. SEM images of (a-e) ZnO/rGO and (f) GO membranes.

Supplementary Figure 25. Water permeance and MB rejection for 30% ZnO/rGO membranes and (50%) ZnO/rGO membranes.

Supplementary Figure 26. Separation performance of ZnO/rGO for mixed dye molecules of methyl blue/methyl orange (MB/MO).

Supplementary Table 1. Gibbs free energy of various reaction on GO surface functional groups.

Supplementary Table 2. Roughness of GO and ZnO/rGO membranes.

Supplementary Table 3. Chemical structures and sizes of five dye molecules used in this work.

Supplementary Table 4. Nanofiltration performance comparation of GO-based membranes.

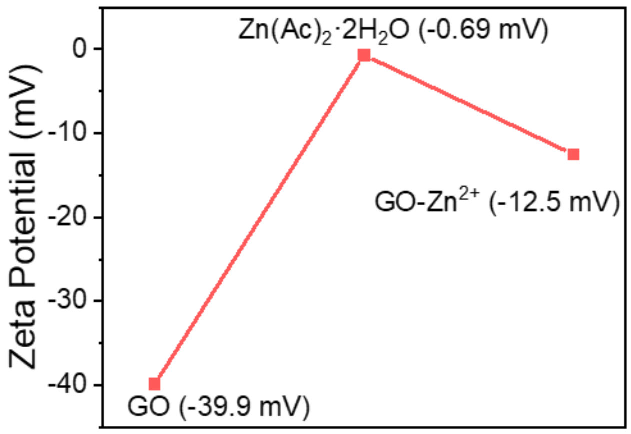

Supplementary Figure 1. Zeta potentials of GO, Zn $(Ac)_2{\cdot}2H_2O$ and GO-Zn $(Ac)_2{\cdot}2H_2O$ mixture.

Additional Analysis: As manifested in Supplementary Figure 1, the surface of graphene oxide (GO) is negatively charged (zeta potential=-40 mV), which originated from the oxygen functional groups located on nanosheets. After mixing GO and neutral $Zn(Ac)_2{\cdot}2H_2O$ (zeta potential=-0.69 mV) in ethylene glycol (EG) solution, the zeta potential shows a positive switch (-12.5 mV), suggesting the adsorption of $[Zn(EG)_2]^{2+}$ on GO surface.

$$Zn(CH_3COO)_2 + HOCH_2CH_2OH \rightarrow [Zn(EG)_2]^{2+} \quad (1)$$

$$HOCH_2CH_2OH \leftrightarrow CH_3CHO + H_2O \quad (2)$$

$$CH_3COO^- + H_2O \leftrightarrow CH_3COOH + OH^- \quad (3)$$

$$[Zn(EG)_2]^{2+} + GO \rightarrow [Zn(EG)_2]^{2+}/GO^* \quad (I)$$

$$[Zn(EG)_2]^{2+}/GO^* + OH^- \rightarrow Zn(OH)_2/rGO^* \quad (II)$$

$$Zn(OH)_2/rGO^* \rightarrow ZnO/rGO^* \quad (III)$$

$$ZnO/rGO^* \rightarrow ZnO/rGO \quad (IV)$$

Supplementary Figure 2. The reaction process of the formation of ZnO/rGO nanocomposites.

Additional analysis: The possible chemical reactions occurred in the system can be expected as Supplementary Figure 2. Briefly, EG is first attract zinc cations (Zn^{2+}) to form stable chelate compounds $[Zn (EG)_2]^{2+}$ (Supplementary Figure 2 (1)). The positively charged $[Zn(EG)_2]^{2+}$ complexes then assemble with negatively charged graphene oxide (GO) (-40 mV) through electrostatic interactions, which corresponds to the adsorption process. During solvothermal process, EG could reversibly react to aldehyde (CH_3CHO) and H_2O (Supplementary Figure 2(2)). The hydroxyl ions (OH^-) produced by hydrolysis of acetate ($CH3COO^-$) (Supplementary Figure 2(3)) stimulate the in-situ transformation of $[Zn (EG)^2]^{2+}$ complex to Zn $(OH)_2$ (Supplementary Figure 2 (I-II)). Meanwhile, GO is also reduced to rGO by EG of reducibility. Finally, ZnO/rGO nanocomposites are formed by the hydrolysis of $Zn(OH)_2$/rGO (Supplementary Figure 2 (III)) and uniformly anchored on rGO.

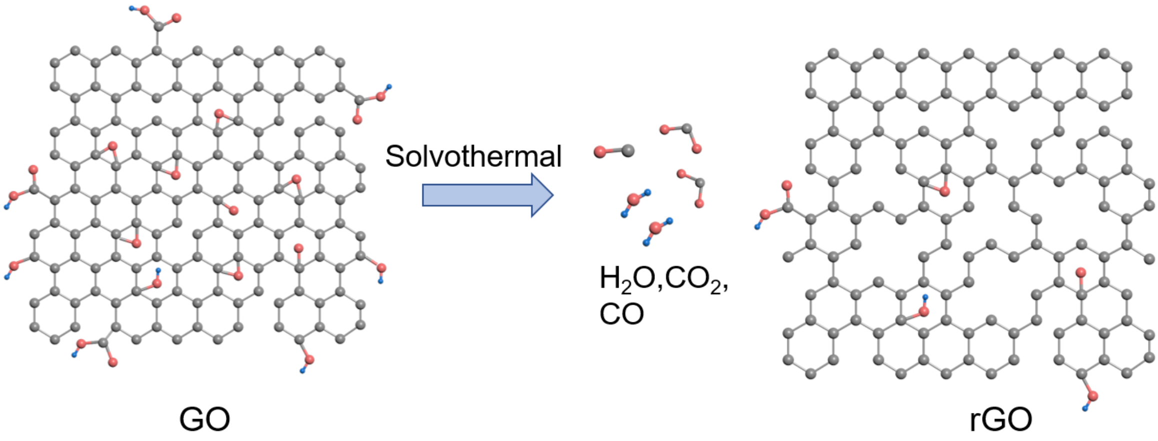

Supplementary Figure 3. Schematic illustration of the reduction of GO.

Additional analysis: GO is covalently functionalized with oxygen containing groups (hydroxyl, epoxide, carbonyl, etc.) on the basal plane and on the edges. During the solvothermal process, carbon monoxide (CO), carbon dioxide (CO_2) and water (H_2O) remove from GO surface leading to the formation of rGO with lots of defects[1, 2].

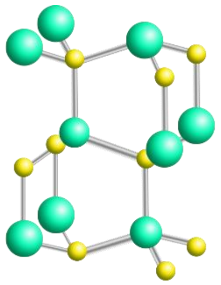

Supplementary Figure 4. Illustration of one ZnO cell (Zn green, O yellow).

Additional analysis: The unit cell volume is approximately 47.6 Å^3. Consequently, ZnO contains approximately 8.4×10^{22} atoms per cm^3. The synthetically produced ZnO with the wurtzite structure may eventually exhibit a crystallographic arrangement different from the pattern established in the crystallographic records[3].

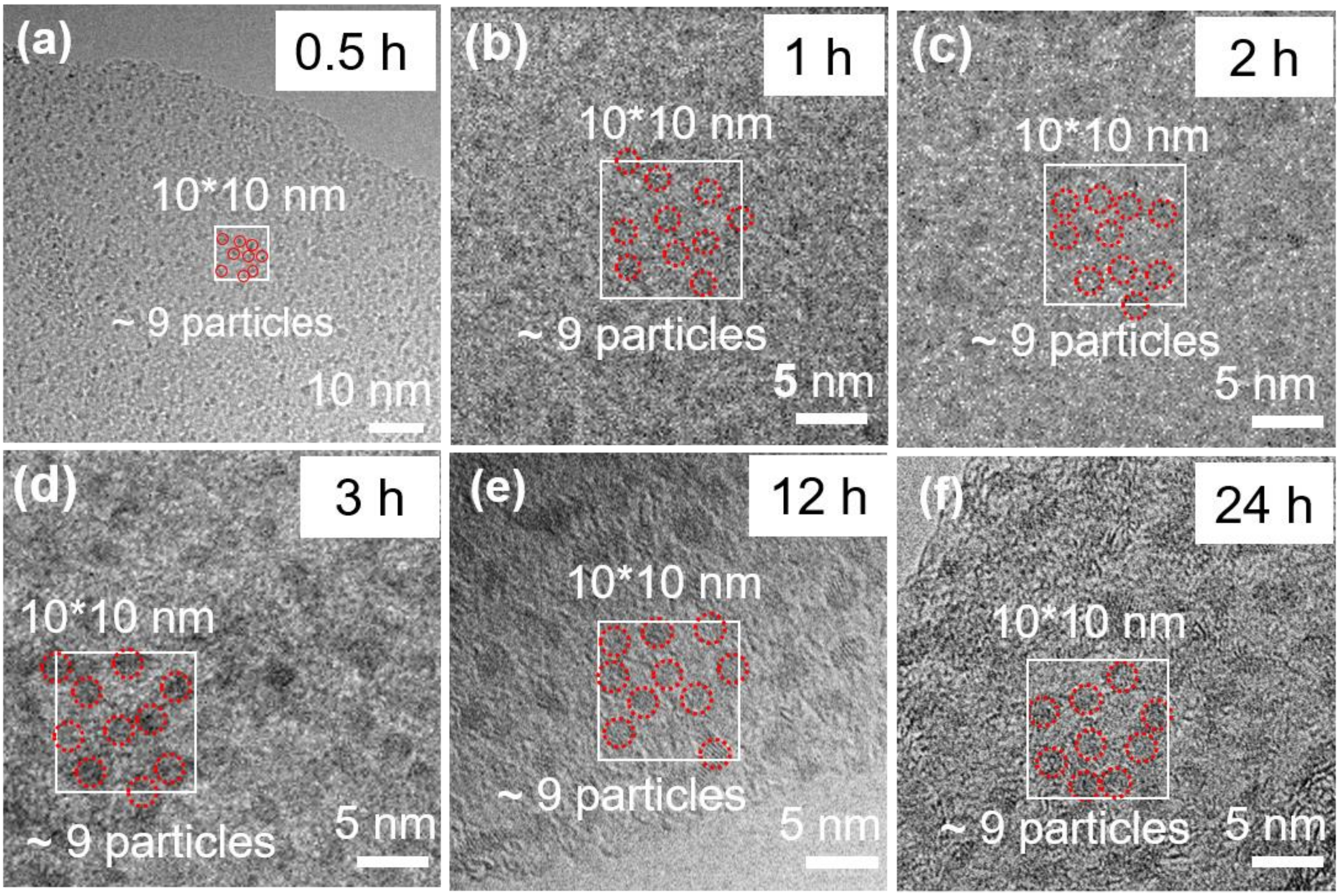

Supplementary Figure 5. TEM images of various samples synthesized at (a) 0.5 h, (b) 1 h, (c) 2 h, (d) 3 h, (e) 12 h and (f) 24 h.

Additional analysis: The particle number density is almost the same of 9 per 10 nm×10 nm for all samples synthesized at different time, indicating that the nucleation occurs simultaneously at GO surface and no new nuclei form during growth process.

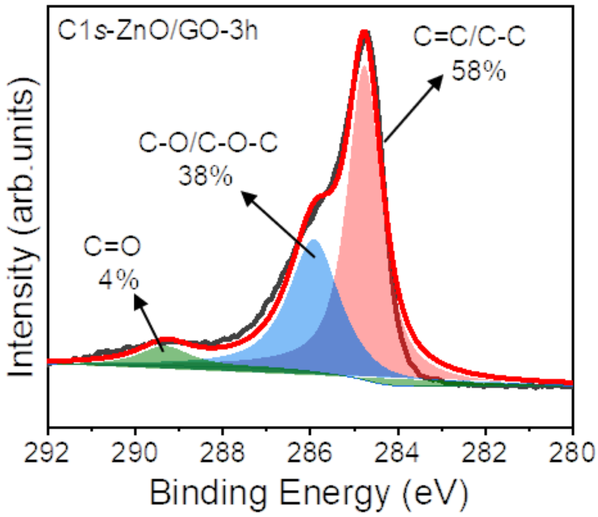

Supplementary Figure 6. High-resolution C 1*s* XPS spectrum curves of ZnO/rGO-3h.

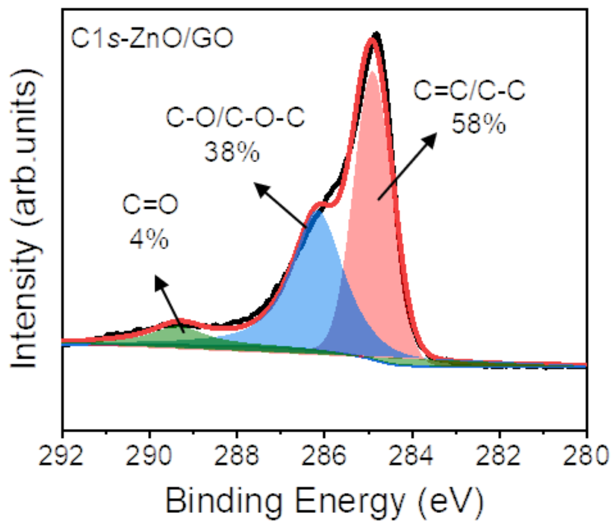

Supplementary Figure 7. High-resolution C 1*s* XPS spectrum curves of ZnO/rGO.

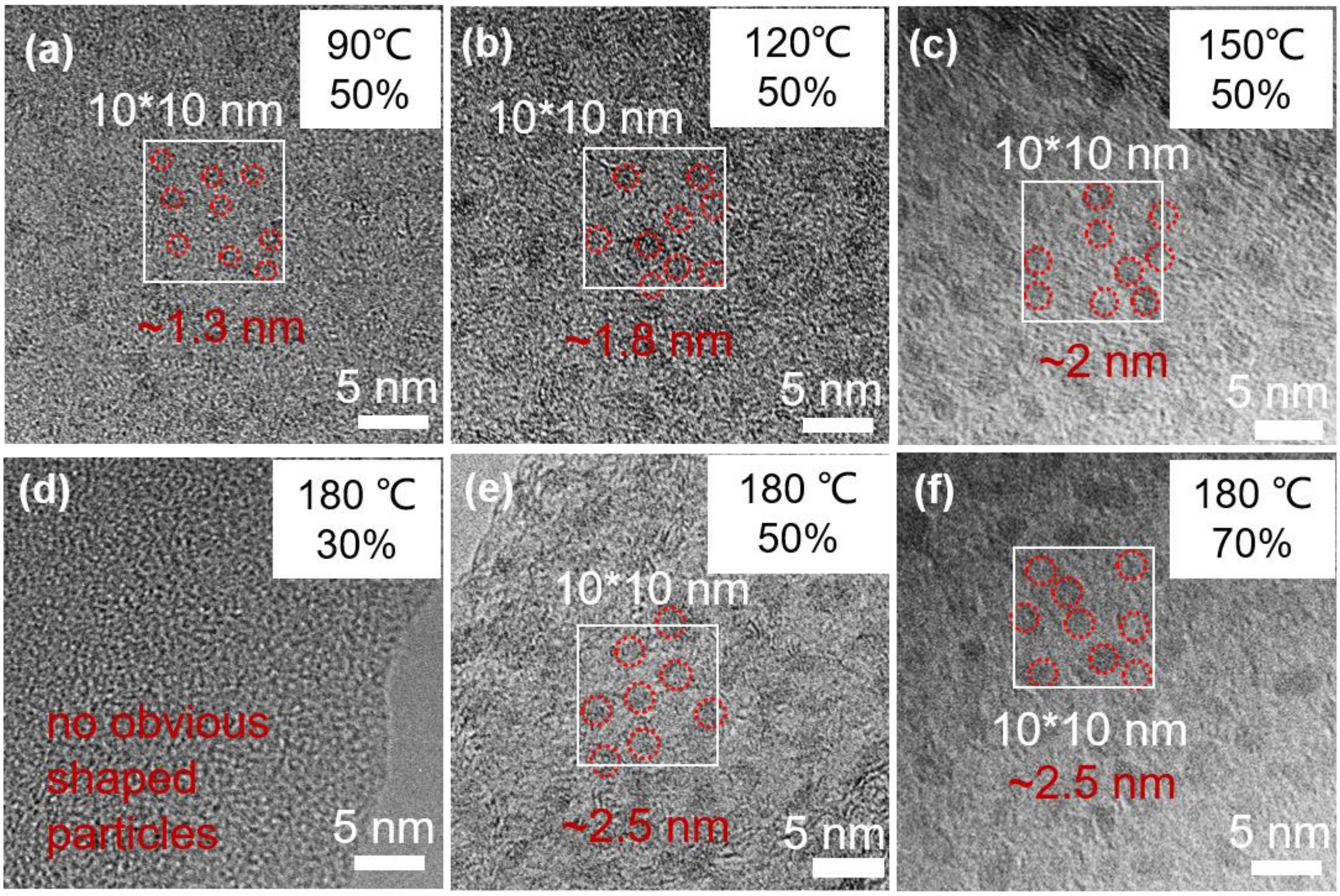

Supplementary Figure 8. HR-TEM images of (a) ZnO/rGO-90°C, (b) ZnO/rGO-120°C, (c) ZnO/rGO-150°C, (d) ZnO/rGO-30%, (e) ZnO/rGO and (e) ZnO/rGO-70%.

Additional Analysis: The samples synthesized at different temperature or with different precursor concentration show the same particle number density of 9 nanoparticles per 10 nm×10 nm.

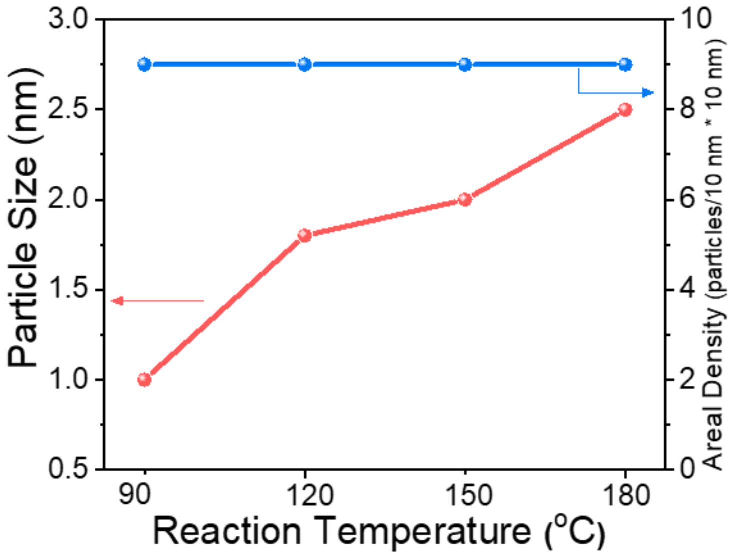

Supplementary Figure 9. Graph of the average particle size and particle number density over temperature.

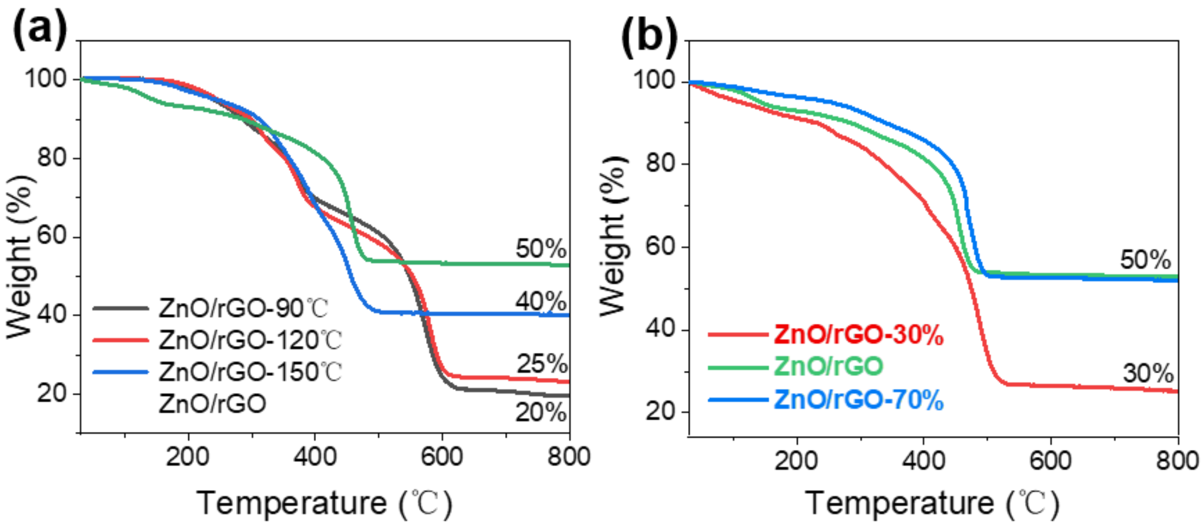

Supplementary Figure 10**.** Thermogravimetric analysis (TGA) curves of samples prepared (a) at various temperature and (b) by various precursor concentration.

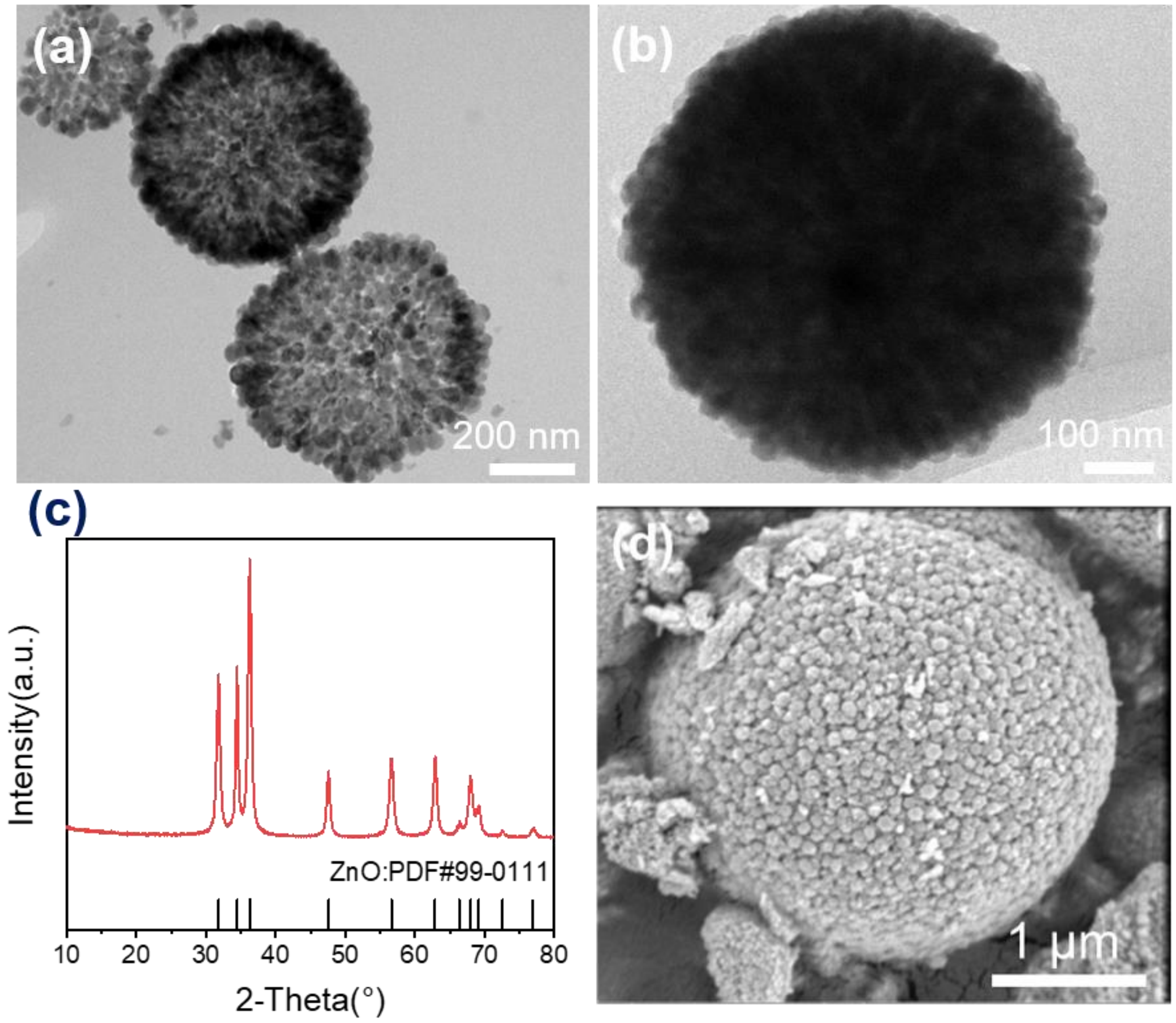

Supplementary Figure 11. (a-b) TEM images, (c) XRD pattern, and SEM image of ZnO.

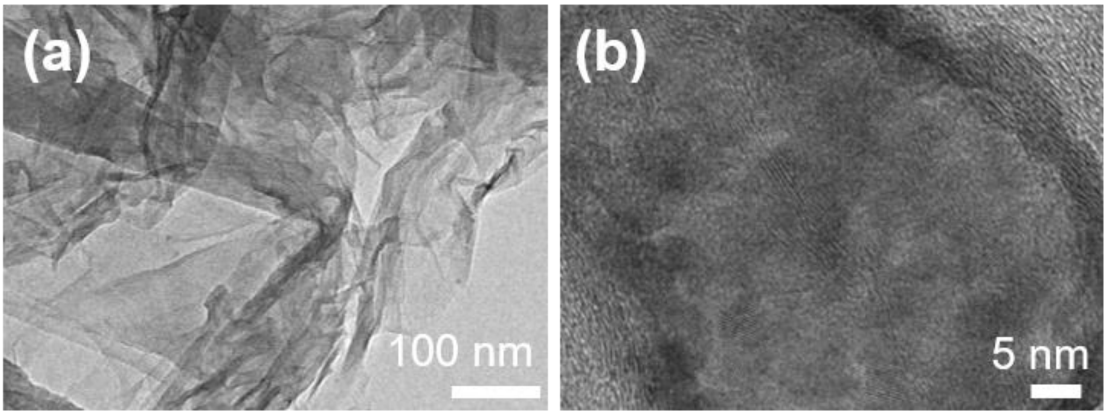

Supplementary Figure 12. (a-b) TEM images of ZnO/rGO-H_2O.

Additional Analysis: The sample ZnO/rGO-H_2O was synthesized using the same process as that of ultrafine ZnO/rGO nanocomposites, except that the solvent EG was changed to deionized water.

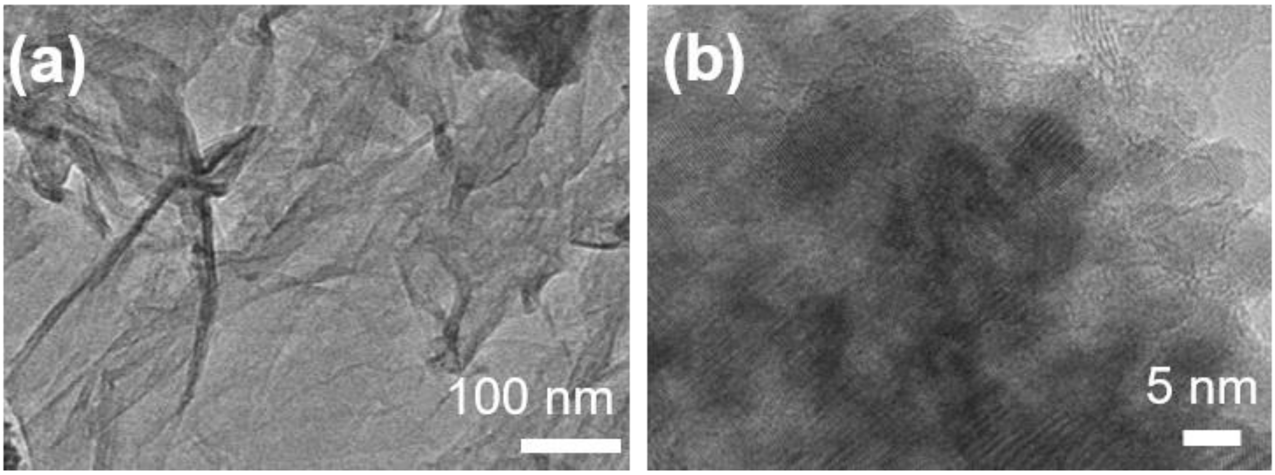

Supplementary Figure 13. (a-b) TEM images of ZnO/rGO-EtOH.

Additional Analysis: The sample ZnO/rGO-EtOH was synthesized using the same process as that of ultrafine ZnO/rGO nanocomposites, except that the solvent EG was changed to EtOH.

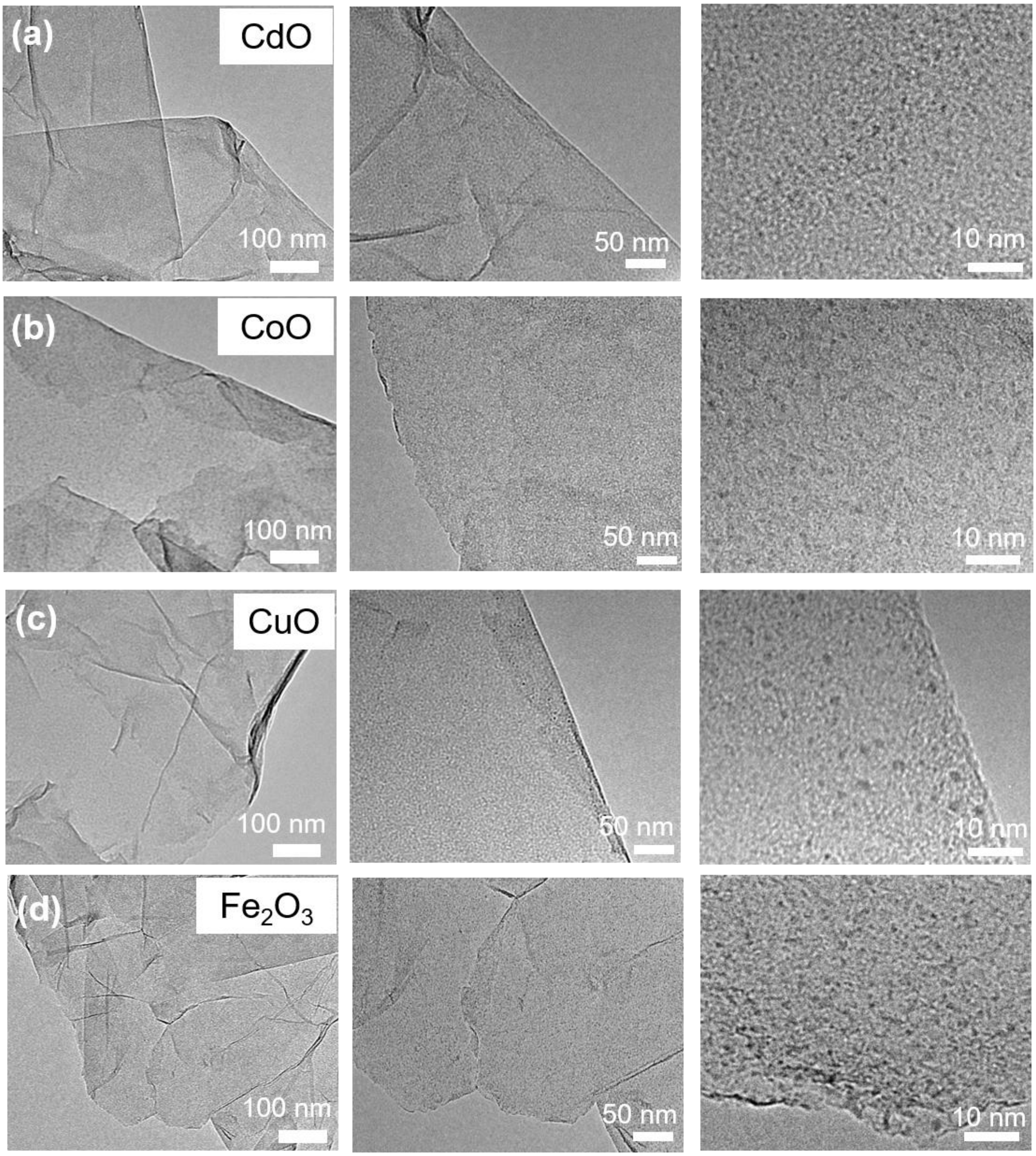

Supplementary Figure 14. TEM images of (a) CdO/rGO, (b) CoO/rGO, (c) CuO/rGO, (d) Fe_2O_3/rGO nanocomposites at different magnifications.

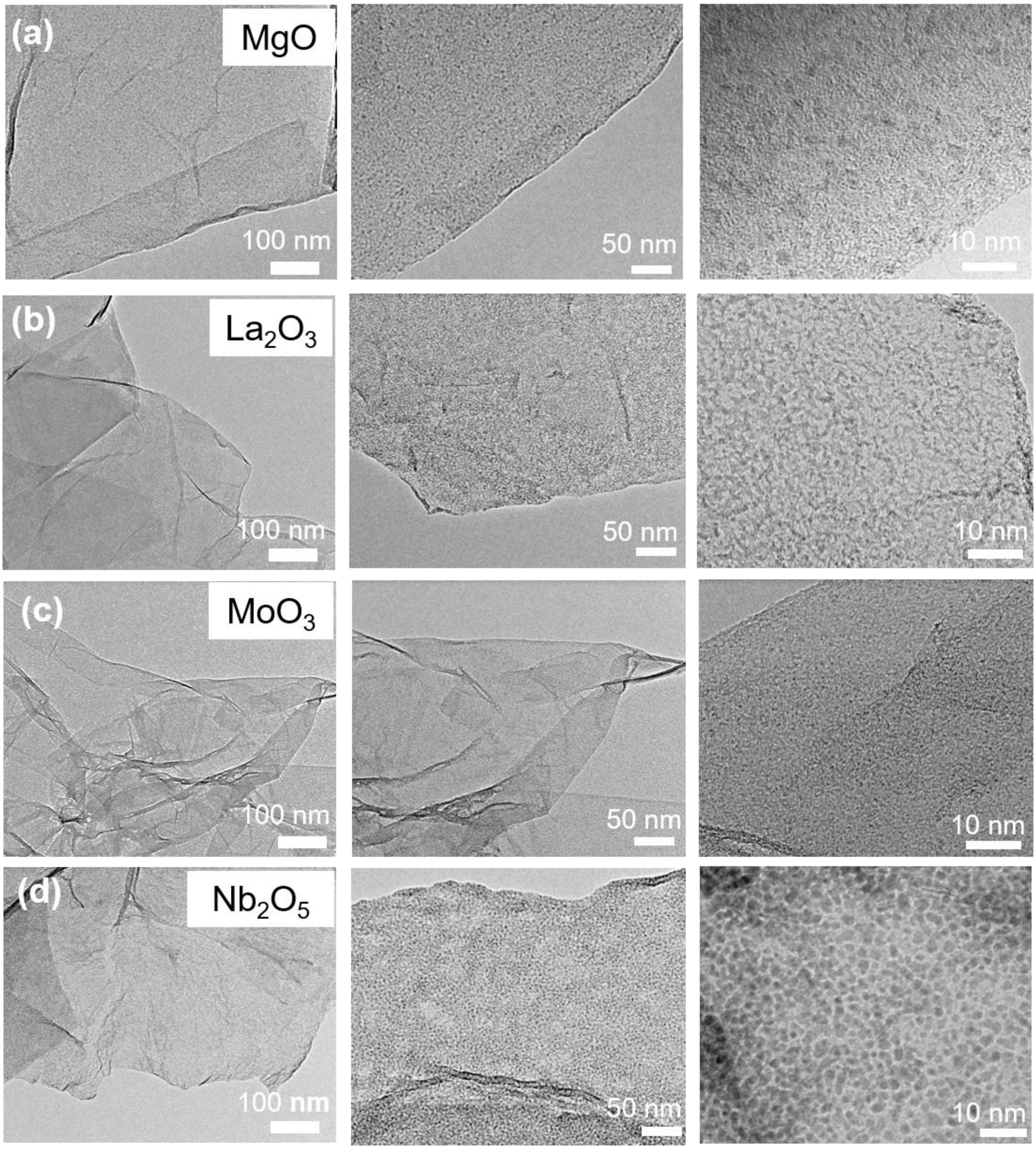

Supplementary Figure 15. TEM images of (a) MgO/rGO, (b) La_2O_3/rGO, (c) MoO_3/rGO, (d) Nb_2O_5/rGO nanocomposites at different magnifications.

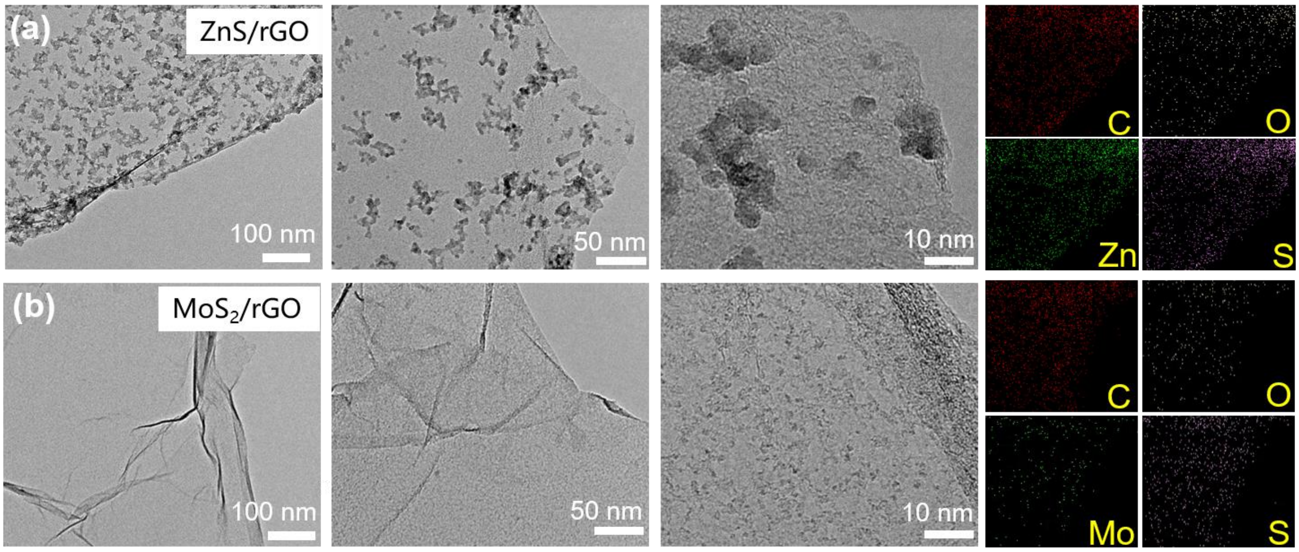

Supplementary Figure 16. TEM images of (a) ZnS/rGO and (b) MoS_2/rGO nanocomposites at different magnifications.

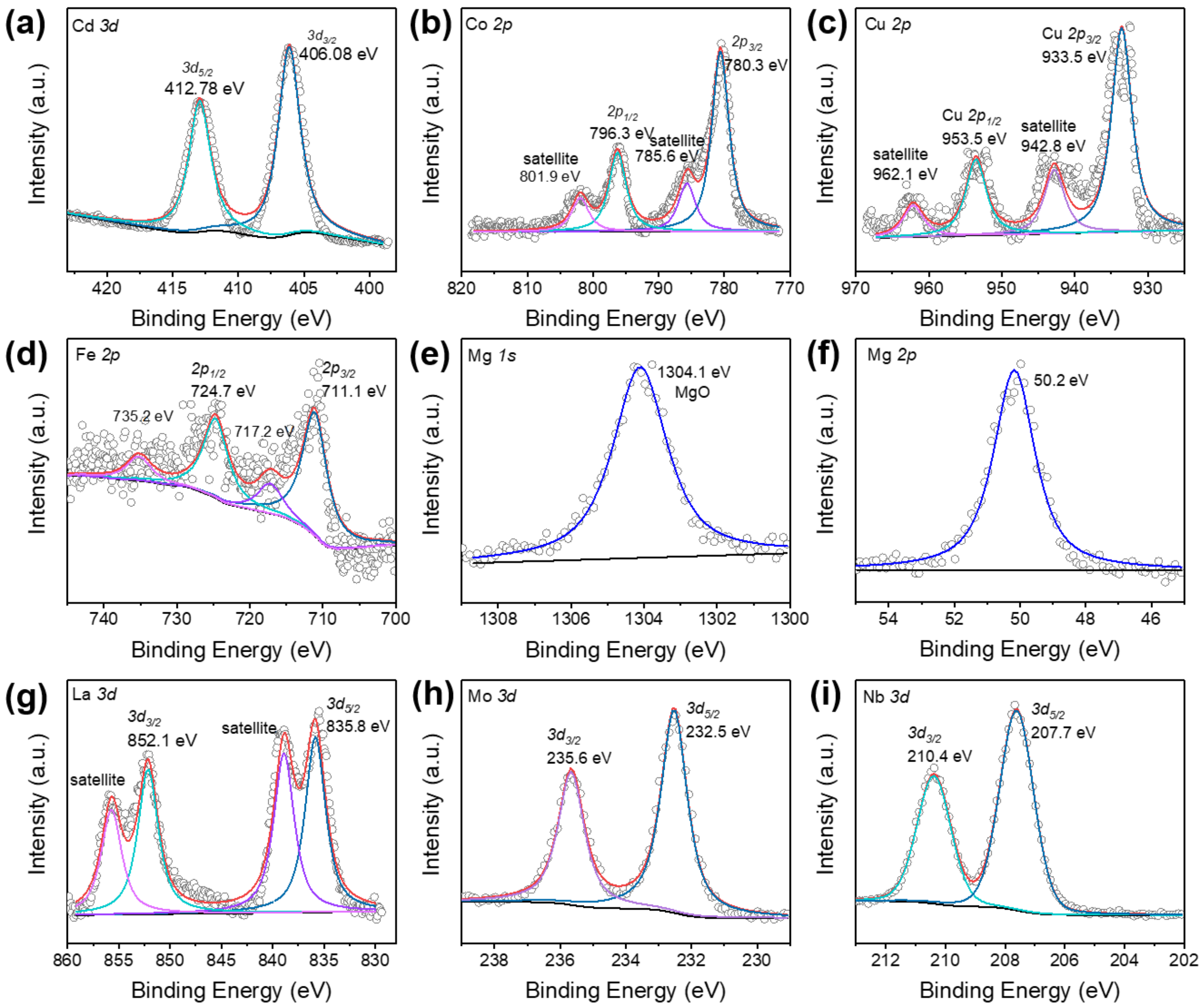

Supplementary Figure 17. (a) Cd 3d XPS pattern of CdO/rGO[4]. (b) Co 2p XPS pattern of CoO/rGO[5]. (c) Cu 2p XPS pattern of CuO/rGO[6]. (d) Fe 2p XPS pattern of Fe_2O_3/rGO[7]. (e) Mg 1s and (f) Mg 2p XPS patterns of MgO/rGO[8]. (g) La 3d XPS pattern of La_2O_3/rGO[9]. (h) Mo 3d XPS pattern of MoO_3/rGO[10]. (i) Nb 3d XPS pattern of Nb_2O_5/rGO[11].

Additional Analysis: The chemical compositions of metal oxide in nanocomposites were confirmed by XPS according to references.

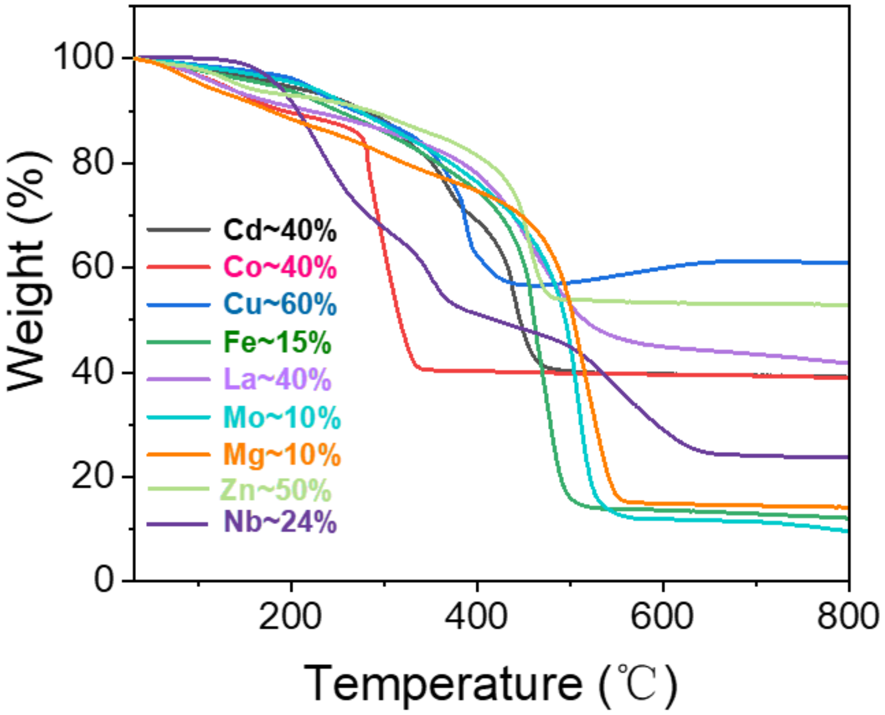

Supplementary Figure 18. Thermogravimetric analysis (TGA) curves of CdO/rGO, CoO/rGO, CuO/rGO, Fe_2O_3/rGO, La_2O_3/rGO, MgO/rGO, MoO_3/rGO, ZnO/rGO and Nb_2O_5/rGO under air atmosphere.

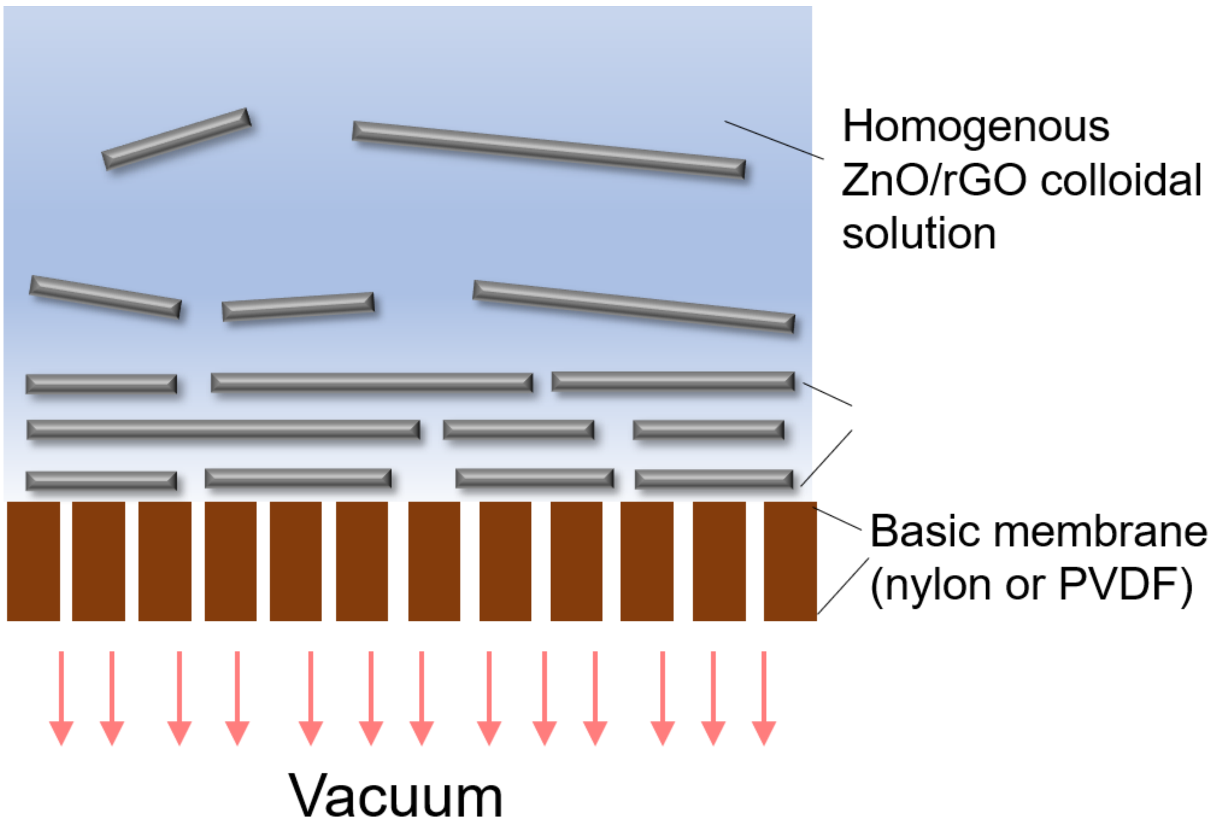

Supplementary Figure 19. Illustration of the preparation of ZnO/rGO membranes through vacuum filtration.

Additional analysis: As illustrated in **Fig. S18**, the vacuum-induced pressure gradient results in dynamic flow which drives rGO nanosheets to deposit on the basic membranes (PVDF). When the surfaces of rGO nanosheets are parallel to the basic membrane surface, the movement is kinetically favorable. While the configuration that rGO nanosheets are perpendicular to the film surface is intrinsically unstable[12]. Also, interaction of π-π stacking promote partial interlocking and overlapping of rGO nanosheets[13, 14].

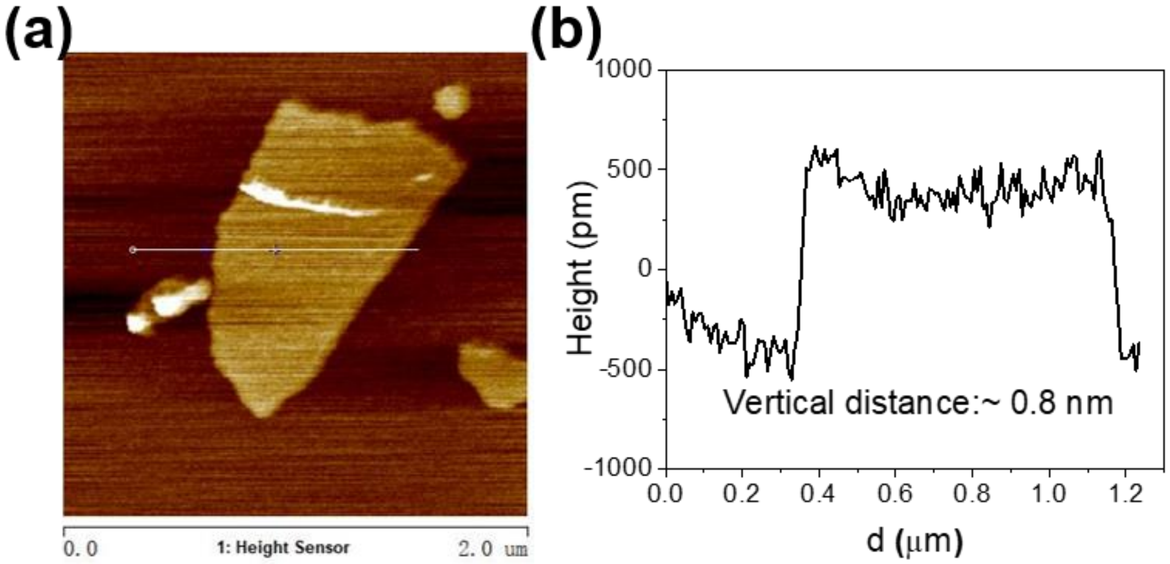

Supplementary Figure 20. (a-b) AFM images of GO.

Additional Analysis: As shown in Supplementary Figure 20 and Fig. 2g, the 2D nanochannel spacing of ZnO/rGO membrane is estimated at ~0.8 nm.

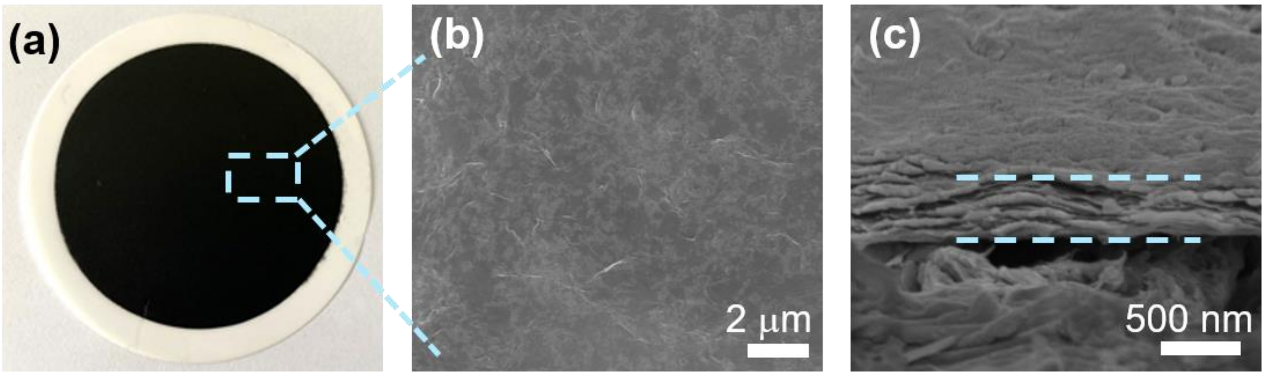

Supplementary Figure 21. (a) Digital photograph, (b) top-view SEM image and (c) cross-section SEM image of ZnO/rGO/nylon membranes.

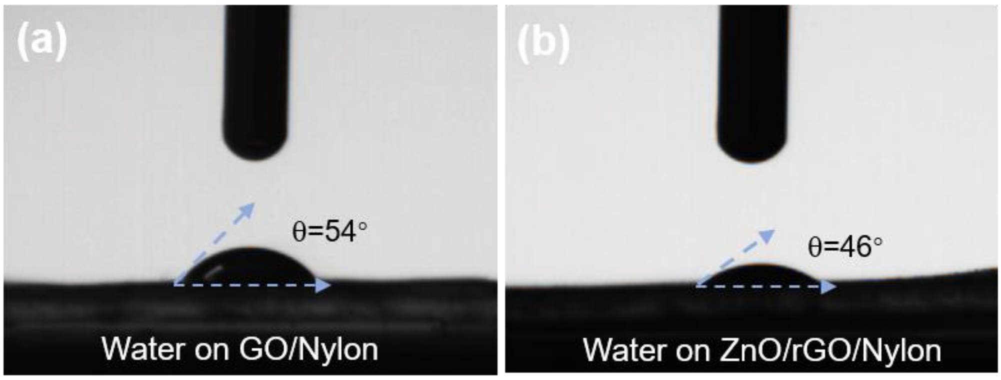

Supplementary Figure 22. Contact angle of water on (a) GO/Nylon and (b) ZnO/rGO/Nylon.

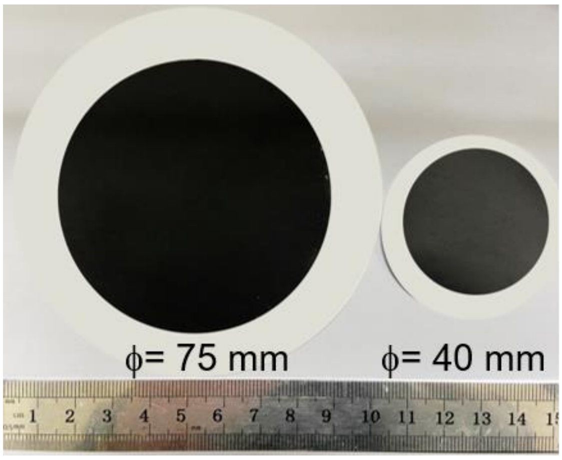

Supplementary Figure 23. Example of a large-area ZnO/rGO/nylon membrane fabricated by vacuum filtration.

Additional Analysis: The large-scale production of ZnO/rGO membranes can be achieved by varying the surface area of nylon membranes and sand core. As shown in **Fig. S22**, the diameter of ZnO/rGO membranes can be broadened from 40 mm to 75 mm.

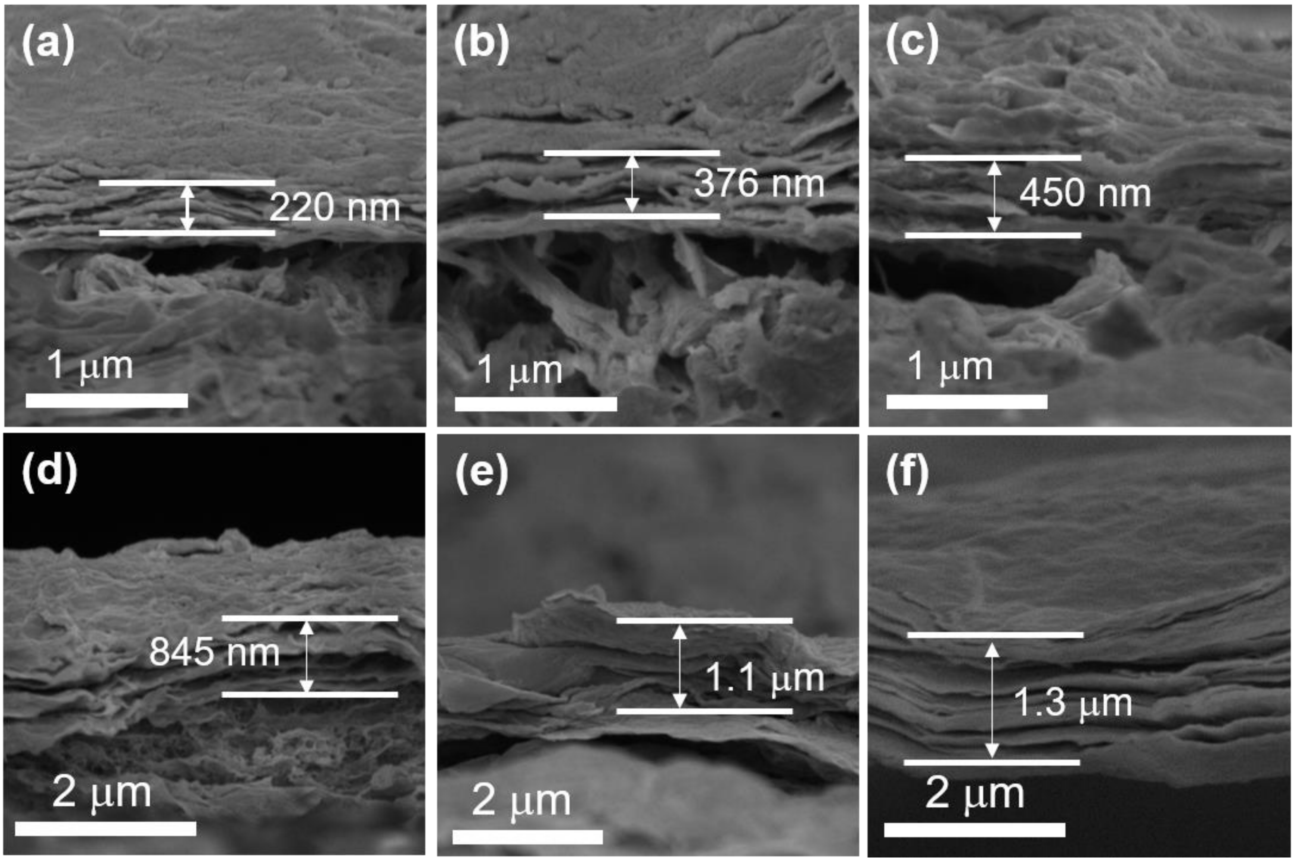

Supplementary Figure 24. SEM images of (a) 0.14 mg cm^{-2}, (b) 0.27 mg cm^{-2}, (c) 0.41 mg cm^{-2}, (d) 0.54 mg cm^{-2}, and (e) 0.68 mg cm^{-2} ZnO/rGO loading amount on nylon membranes. (f) 0.54 mg cm^{-2} loading amount of GO on nylon membrane.

Additional Analysis: From cross-section SEM image (Fig. S18), GO membrane presents a uniform laminar structure, while the layers in ZnO/rGO membranes were wrinkled with larger interlayer spacing.

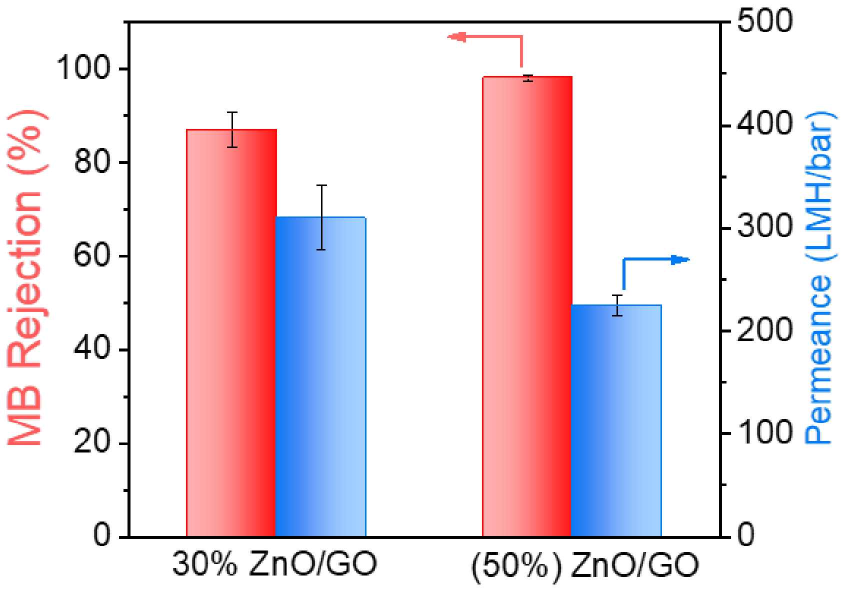

Supplementary Figure 25. Water permeance and MB rejection for 30% ZnO/rGO membranes and (50%) ZnO/rGO membranes. Error bars represent standard deviations from measurements of three different samples.

Additional Analysis: GO-based membranes are great promising for advanced nanofiltration in water treatments, but there is a trade-off between water flux and selectivity. Large interlayer spacing leads to high water flux and low rejection, while small interlayer spacing leads to low water flux and high rejection. To coordinate the water flux and dye selectivity of ZnO/rGO membranes. We have prepared ZnO/rGO nanocomposites with different ZnO contents in nanocomposites. As shown in **Fig. S23**, the 30% ZnO/GO membranes at the same mass loadings are prepared, which exhibits only 87% selectivity of MB, though the water flux reaches 310 L m^{-2} h^{-1} bar^{-1}. In practical application, selectivity of dye molecules shall be at least 95%. Therefore, we choose the (50%) ZnO/rGO membranes for further test.

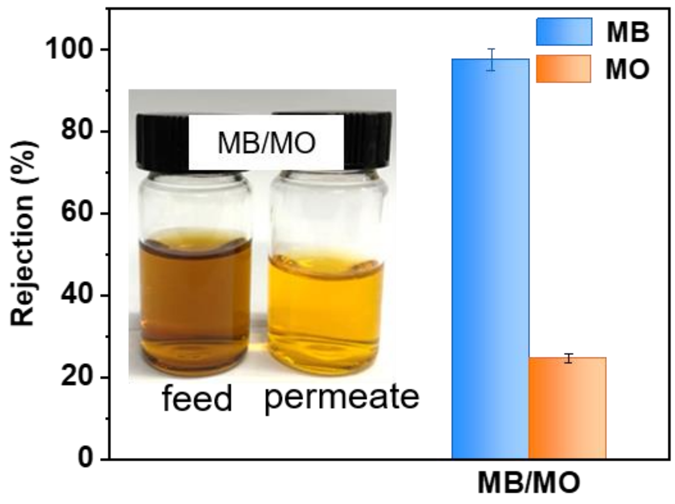

Supplementary Figure 26. Separation performance of ZnO/rGO for mixed dye molecules of methyl blue/methyl orange (MB/MO). Error bars represent standard deviations from measurements of three different samples.

Supplementary Table 1. Gibbs free energy of various reaction on GO surface functional groups.

Functional groups	ΔG (I) /eV	ΔG (II) /eV	ΔG (III) /eV	ΔG_{total} /eV
-OH	-0.349	-7.376	1.292	-6.433
-COC	-1.089	-6.868	0.958	-6.999
-COOH	-1.036	-7.282	1.710	-6.6.08
-CO	-1.072	-6.733	0.046	-7.759
without GO		-6.412	1.539	-4.873

*ΔG (I): Gibbs free energy of reaction adsorption of Zn^{2+} onto GO surface;

ΔG (II): Gibbs free energy of reaction $Zn^{2+}* \rightarrow Zn(OH)_2*$;

ΔG (III): Gibbs free energy of reaction $Zn(OH)_2* \rightarrow ZnO*$;

ΔG_{total}: Gibbs free energy of the whole reaction.

Supplementary Table 2. Roughness of GO and ZnO/rGO membranes.

Membranes	Ra (nm)	Rq (nm)
GO	0.286	0.414
ZnO/rGO	3.95	4.61

Supplementary Table 3. Nanofiltration performance comparation of GO-based membranes.

Membranes	Loading mass (mg cm^{-2})	Thickness (μm)	Pressure (bar)	MB Rejection (%)	Water Permeance (L m^{-2} h^{-1} bar^{-1})	Ref
GO	0.54	1.4	1	98.2	20	This work
ZnO/rGO	0.54	0.845	1	98.1	225	This work
PEI-GO/PAA/PVA/GA			5	99.3	0.87	[15]
PDDA/GO		0.01	5	99.2	6.42	[16]
PAN/GO			6	100	5.5	[17]
GO/OCNTs			3	99.3	7.24	[18]
ZIF-8@GO		0.105	1	100	49.81	[19]
GO/g-C3N4	0.0247		1	100	76.6	[20]
C-BCGO-Au-stc-M		4	5	24.4	32.4	[21]
GO/COF		2.7	1	99.53	58.58	[22]
DES/GO	0.0035		3	96.7	21	[23]
PVDF/SDS-GO/TiO_2		18.6	2	92.79	4.63	[24]

Supplementary Table 4. Chemical structures and sizes of five dye molecules used in this work[25].

Organic dye	Molecular weight (g mol^{-1})	Solute Charge	Molecular size (nm*nm)	Chemical structure
Methylene Blue (MLB)	319.85	+	1.25*0.51	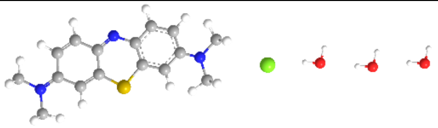
Methyl Orange (MO)	327	-	1.13*0.42	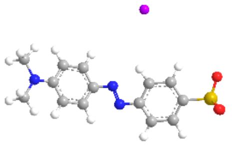
Chrome Black T (CBT)	461	-	1.55*0.88	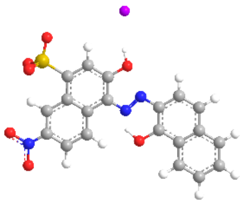
Rhodamine B (RhB)	479	+	1.13*1.20	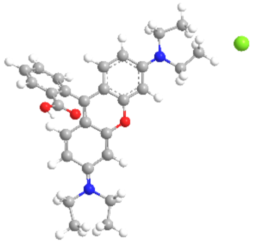
Congo Red (CR)	696	-	2.56*0.73	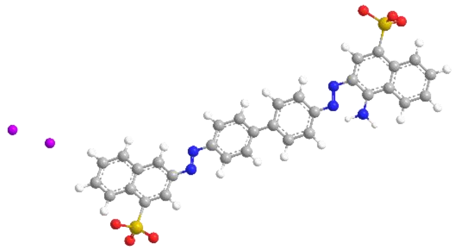
Methyl Blue (MB)	799.8	-	2.36*1.74	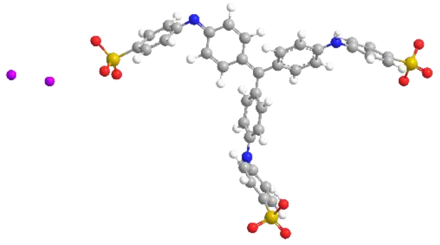
Evans Blue (EB)	960	-	3.1*1.3	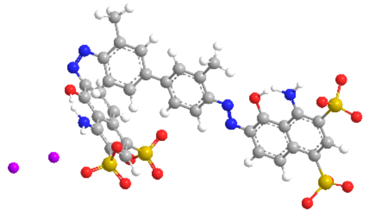

* C: grey balls; H: white balls; S: yellow balls; O: red balls; N: blue balls; Cl: green balls. The

molecular sizes were calculated using ChemOffice2014.

Supplementary References

1. Pei, S. and H.-M. Cheng, *The reduction of graphene oxide.* Carbon, 2012. **50**(9): p. 3210-3228.
2. Bagri, A., et al., *Structural evolution during the reduction of chemically derived graphene oxide.* Nature chemistry, 2010. **2**(7): p. 581-587.
3. CrystalMaker. *Structure Type 014: ZnO (wurtzite)*. Available from: http://som.web.cmu.edu/structures/S014-ZnO.html.
4. Saravanan, R., et al., *ZnO/Ag/CdO nanocomposite for visible light-induced photocatalytic degradation of industrial textile effluents.* J Colloid Interface Sci, 2015. **452**: p. 126-133.
5. Chang, L., et al., *Hierarchical CoO microflower film with excellent electrochemical lithium/sodium storage performance.* J. Mater. Chem. A, 2017. **5**(39): p. 20892-20902.
6. Sun, L., et al., *Nitrogen‐Doped Carbon‐Coated CuO‐In2O3 p‐n Heterojunction for Remarkable Photocatalytic Hydrogen Evolution.* Advanced Energy Materials, 2019. **9**(48).
7. Wang, Q. and Y. Wang, *Overcoming the Limiting Step of Fe2O3 Reduction via in Situ Sulfide Modification.* ACS Appl Mater Interfaces, 2016. **8**(16): p. 10334-42.
8. Ding, G., et al., *Waste to Wealth: Exhausted Nitrogen-Doped Mesoporous Carbon/MgO Desulfurizers Turned to High-Sulfur-Loading Composite Cathodes for Li-S Batteries.* ACS Appl Mater Interfaces, 2019. **11**(21): p. 19096-19103.
9. Cheng, Z., et al., *La2O3 modified silica-pillared clays supported PtOx nanocrystalline catalysts for catalytic combustion of benzene.* Chemical Engineering Journal, 2020. **392**.
10. Kawase, M., et al., *Elucidation of the enhanced photoactivity of melon calcined with MoO3.* Applied Catalysis B: Environmental, 2020. **273**.
11. Huang, Q., et al., *Rational cooperativity of nanospace confinement and rapid catalysis via hollow carbon nanospheres@ Nb-based inorganics for high-rate Li-S batteries.* Chemical Engineering Journal, 2021. **411**: p. 128504.
12. Eda, G., G. Fanchini, and M. Chhowalla, *Large-area ultrathin films of reduced graphene oxide as a transparent and flexible electronic material.* Nature nanotechnology, 2008. **3**(5): p. 270-274.
13. Joshi, R.K., et al., *Precise and ultrafast molecular sieving through graphene oxide membranes.* Science, 2014. **343**(6172): p. 752-4.
14. Karan, S., et al., *Ultrafast viscous permeation of organic solvents through diamond-like carbon nanosheets.* Science, 2012. **335**(6067): p. 444-447.
15. Wang, N., et al., *Self-assembly of graphene oxide and polyelectrolyte complex nanohybrid membranes for nanofiltration and pervaporation.* Chemical Engineering Journal, 2012. **213**: p. 318-329.
16. Wang, L., et al., *Layer-by-layer self-assembly of polycation/GO nanofiltration membrane with enhanced stability and fouling resistance.* Separation and Purification Technology, 2016. **160**: p. 123-131.
17. Qiu, Z., X. Ji, and C. He, *Fabrication of a loose nanofiltration candidate from Polyacrylonitrile/Graphene oxide hybrid membrane via thermally induced phase separation.* J Hazard Mater, 2018. **360**: p. 122-131.

18. Kang, H., et al., *Sandwich morphology and superior dye-removal performances for nanofiltration membranes self-assemblied via graphene oxide and carbon nanotubes.* Applied Surface Science, 2018. **428**: p. 990-999.
19. Zhang, W.H., et al., *Graphene oxide membranes with stable porous structure for ultrafast water transport.* Nat Nanotechnol, 2021. **16**(3): p. 337-343.
20. Wu, Y., et al., *2D Heterostructured Nanofluidic Channels for Enhanced Desalination Performance of Graphene Oxide Membranes.* ACS Nano, 2021. **15**(4): p. 7586-7595.
21. Zhong, Y., et al., *Graphene oxide modified membrane for highly efficient wastewater treatment by dynamic combination of nanofiltration and catalysis.* J Hazard Mater, 2020. **397**: p. 122774.
22. Chen, L., et al., *High performance hierarchically nanostructured graphene oxide/covalent organic framework hybrid membranes for stable organic solvent nanofiltration.* Applied Materials Today, 2020. **20**.
23. Mehrabi, N., H. Lin, and N. Aich, *Deep eutectic solvent functionalized graphene oxide nanofiltration membranes with superior water permeance and dye desalination performance.* Chemical Engineering Journal, 2021. **412**.
24. Suriani, A.B., et al., *Incorporation of Electrochemically Exfoliated Graphene Oxide and TiO2 into Polyvinylidene Fluoride-Based Nanofiltration Membrane for Dye Rejection.* Water, Air, & Soil Pollution, 2019. **230**(8).
25. Fan, H., et al., *High‐flux membranes based on the covalent organic framework COF‐LZU1 for selective dye separation by nanofiltration.* Angewandte Chemie International Edition, 2018. **57**(15): p. 4083-4087.
